# Metal Ion-Specific Modulation of Network Connectivity and Defects in Poly(ethylene glycol)–Peptide Conjugate Assemblies and Hydrogels

**DOI:** 10.1021/acs.chemmater.5c02542

**Published:** 2026-01-15

**Authors:** Mostafa Ahmadi, Kamila Wittek, Hanna Sophie Rieger, Marius Thomas, Lars Hartmann, Pol Besenius, Sebastian Seiffert

**Affiliations:** Department of Chemistry, Johannes Gutenberg-Universität Mainz, Duesbergweg 10-14, Mainz D-55128, Germany

## Abstract

Self-assembling peptide–polymer conjugates offer a versatile platform to engineer nanostructures with tunable morphology and functions. Here we show that alternating phenylalanine–histidine pentapeptide units, conjugated to a short linear poly­(ethylene glycol), show pH-induced assembly into β-sheet nanofibers that act as multifunctional cross-links in the resulting hydrogels. Circular dichroism spectra demonstrate that the self-assembly is enthalpy driven at low concentrations, while rheological results suggest that the network connectivity at high concentrations is compromised by the entropic penalty of chain stretching. Metal ions (Co^2+^, Ni^2+^, Cu^2+^, Zn^2+^) enhance secondary structures, with coordination geometry-dependent change of the global assembly. Common impacts of metal coordination include orders-of-magnitude higher network stability, an expanded linear viscoelastic region, and improved network recovery, all indicative of the fast association of metal complexes. Collectively, these results highlight the role of metal ions in tuning supramolecular packing, nanofiber morphology, and consequent hydrogel mechanics in peptide–polymer conjugate assemblies and their role in modulating structure–dynamics–property relationships for applications as stimuli-responsive biomaterials.

## Graphical abstract

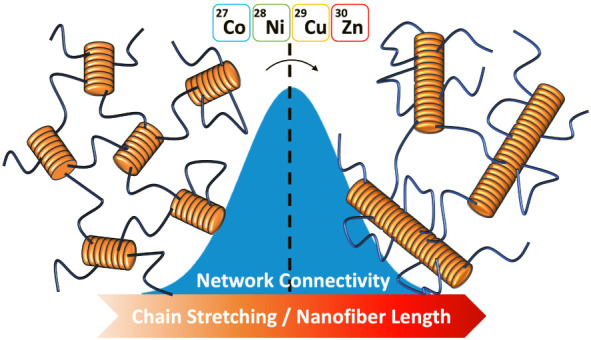

## 1 Introduction

Protein unfolding and aggregation are supramolecular self-assembly processes that are associated with a range of human diseases, including Alzheimer, Parkinson, and type II diabetes.^1^ Additionally, aggregation poses challenges for the development of therapeutic proteins and peptides, as it can induce adverse immune reactions or decrease drug efficiency. Aggregation-prone regions (APRs) are typically short hydrophobic peptides, otherwise buried inside a hydrophilic pocket, which become exposed upon protein unfolding, and tend to form secondary assemblies by interacting with alike hydrophobic segments.^1^ Several types of assemblies have been identified, determined by peptide characteristics such as length, composition, and sequence.^1–4^ The main classes of peptide secondary structures include β-sheets and α-helices, which can give rise to amyloid fibrils and coiled-coil assemblies, respectively, driven by hydrogen bonding, hydrophobic effects, bulky aromatic residues, and π–π stacking.^1,2^ Because of their inherent stability, these secondary structures occur widely in natural systems, for example in spider silk, and have consequently inspired chemists to design and control robust supramolecular assemblies from weak interactions.^5^ Therefore, a detailed study of how such secondary structures form is crucial, for realizing possible application of the resulting biocompatible systems in food technology, therapeutics, and (bio)­materials.^1,5^

Advances in solid-phase peptide synthesis and ring-opening polymerization methods have enabled the development of diverse ordered structures, achieved through variations in amino acid sequences and their conjugation with polymers.^6^ Nevertheless, prediction of secondary structures is not straightforward, as the same amino acid can promote different assemblies depending on its close neighboring and even long-range interactions, besides the environmental factors like pH, temperature, and solvent interactions.^7^ When embedded in polymer structures, their intramolecular assembly enables the formation of nanostructures,^8–10^ whereas their intermolecular assembly results in network formation.^10–13^ As such, tuning the type and extent of assembly not only results in the manipulation of mechanical properties, but also provides stimuli-responsiveness.^2,14^

Several external factors have been reported to manipulate the assembly of peptides and their polymer conjugates. Specifically, temperature can weaken hydrogen bonding, while redox reactions and pH can (de)­protonate ionizable amino acids, thereby shifting the hydrophobic/hydrophilic balance allowing control over the assembly process.^15^ Nevertheless, one of the most versatile and less explored triggers is the complexation of peptide sequences with metal ions.^16–18^ Histidine (His) and cysteine residues readily form strong coordination bonds with metal ions, while the phenolic hydroxyl of tyrosine and the terminal amine of lysine can act as weaker ligands under suitable conditions.^19,20^ In addition, backbone carbonyl groups and amide functionalities may contribute weakly to metal coordination, a feature common to all amino acid units. Accordingly, the mechanical toughness and self-healing behavior of mussel byssus threads are believed to arise from reversibly breakable metal coordination cross-links embedded in His-rich protein domains (HRD).^17,21^ Moreover, transition metal ions have long been associated with the aggregation of β-amyloid plaques in Alzheimer’s disease, promoting neuronal dysfunction.^22^

While the effect of the coordination geometry of metal ions on the characteristics of discrete metal–ligand complexes is rather predictable,^23–25^ their impact on secondary structure formation is quite complex.^9,26–28^ Holten-Andersen et al. have shown that His-terminated tetra-arm poly­(ethylene glycol) (tetraPEG) chains can form a percolated network only upon complexation with transition metal ions.^29,30^ However, Harrington et al. have demonstrated that tetraPEG functionalized with rather long HRD peptide sequences undergoes rapid pH-triggered gelation through the self-organization of peptide groups into β-amyloid structures, even in the absence of metal ions.^21,31^ Interestingly, metal ions exert contrasting influences on this assembly, with Zn^2+^ stabilizing the tetraPEG–HRD hydrogels and Ni^2+^ functioning as an inhibitor of cross-link formation. This likely originates from the difference in the coordination geometry preference of metal ions; the octahedral preference of Ni^2+^, unlike the tetrahedral preference of Zn^2+^, supposedly competes with the packing of β-sheet-ordered domains.^27,28,32^ Similarly, tetraPEG chains functionalized with complementary peptides, consisting of heptad sequences capable of forming a superhelix coiled-coil assembly, could form a percolated network in the absence of metal ions.^33^ Further association of His groups, embedded at the solvent-exposed positions of the heptad, with Ni^2+^ could stabilize the network structure,^26^ whereas superhelices would aggregate in irregular structures upon intermolecular coordination with Zn^2+^.^34^ Other peptide sequences have similarly shown metal ion-specific distinct secondary structures.^9,18,20,35–37^ Diverse effects of different metal ions on the formation of higher-order cross-links through mineralization are also reported in other systems. The HRD of the worm jaw can form hydrogels that significantly stiffen upon exposure to metal ions. Zn^2+^ cations are reported to exclusively sclerotize the hydrogel through the formation of coordinative cross-links.^38^ We have also reported on the diverse affinity of various metal ions for the spontaneous formation of metal nanoparticles in polymeric systems with inherent reducing affinity.^39,40^ The complex effect of metal ions, combined with the diversity in the number, composition, and sequence of various amino acids in peptides, results in materials with quite complex structures and behavior. The number and placement of His, for instance, are reported to be effective in complexation. Two His groups either in positions *i* and *i* + 2 or *i* and *i* + 5 of a hexapeptide are contributing more to the overall binding than the other possible sequence combinations.^41^ Therefore, systematic investigations into the impact of various metal ions in the assembly pathway of synthetic peptides and their polymer conjugates remain scarce but have tremendous potential for controlling higher order structures, with potential applications in biomaterials development.

To address this gap, herein we study the effect of various metal ions on an amphiphilic peptide–polymer conjugate system, containing the metal-coordinating His and hydrophobic phenylalanine (Phe) groups in the oligopeptide domain. We employ a short alternating pentad sequence connected to the termini of a linear hydrophilic PEG chain to mimic natural protein structures with short APRs. While the system shows an intrinsic affinity for forming β-sheet nanofibers at neutral pH conditions, the addition of various metal ions shows distinct effects on the secondary structure and consequent properties of the hydrogels, as revealed by rheological measurements, spectroscopy, and microscopy. These results can not only help in understanding the role of metal ions in peptide aggregation but also can be exploited for designing advanced stimuli-responsive (bio)­materials based on modulating secondary structure.

## 2 Experimental Section

### 2.1 Materials and Synthesis

Details of the chemicals used, synthesis, and characterizations are explained in Section S1 of SI. Briefly, the pentapeptide sequence of alternating Phe and His moieties, with the two His side chains protected with trityl groups, was synthesized using conventional solid-phase peptide synthesis. The PEG–peptide conjugate was obtained through PyBOP-mediated amidation of a diamine-modified PEG, followed by deprotection of His groups under acidic conditions, as briefly illustrated in Figure 1a. Updated characterization results are provided in the SI.

**Figure 1:**
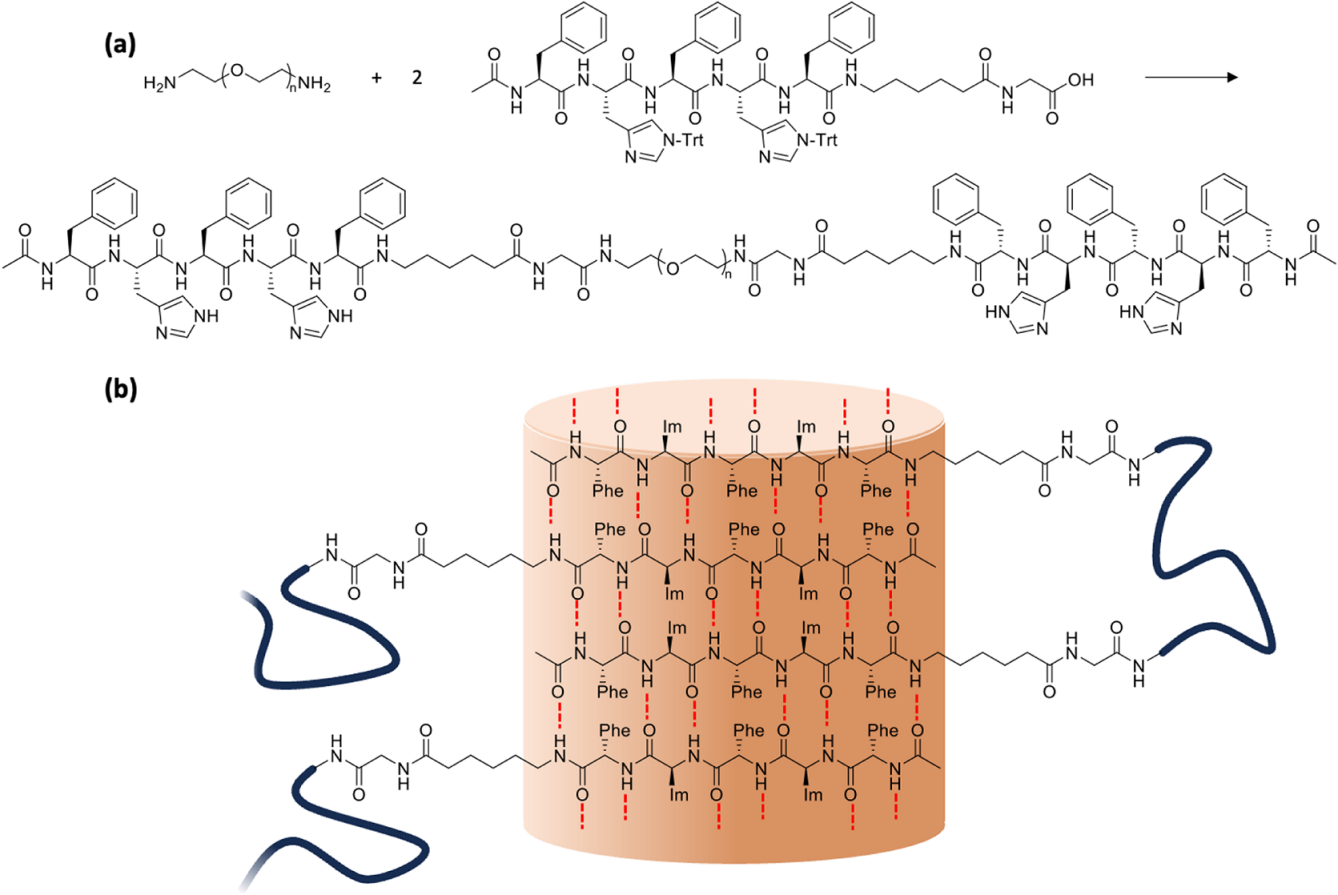
(a) Synthesis scheme for the conjugation of the peptide sequence and PEG diamine. (b) Hydrogen bonding-driven assembly of the peptide sequence in β-sheet structures.

### 2.2 Characterization

The pH values of all samples were measured using an MI-410 micro pH electrode probe. The pH meter was calibrated using Mettler-Toledo certified buffer solutions at pH 4, 7, and 10. First the conjugate solution was prepared in PBS buffer at a pH of 3 at the desired concentration. Then, the pH was further adjusted between 2 and 3 using a 1 N HCl solution to obtain a transparent solution. The metal ion solution was subsequently added if needed and mixed thoroughly. Subsequently, the pH was adjusted to the desired value using a 1 N NaOH solution. The pH was further checked and adjusted the next day. The negligible decrease in pH over time could be due to the time-consuming diffusion of OH ions and the establishment of the equilibrium structure.

The conjugate solutions at concentrations between 1 and 3 wt % formed a turbid hydrogel at pH values above 6. Rheological measurements were carried out on a stress-controlled Anton Paar Physica MCR 301 rheometer with a 25 mm cone–plate geometry. A few drops of silicone oil were placed around the sample to avoid evaporation. The standard Anton Paar solvent trap was used to further minimize the solvent evaporation. Rheological measurements consisted of (I) temperature adjustment to 10 °C (10 min), followed by a three-segment oscillatory thermal treatment (γ = 0.01, ω = 10 rad s^–1^) including a linear heating ramp up to 50 °C (0.02 C min^–1^), an isothermal time sweep at 50 °C (10 min), and a linear cooling ramp back to 10 °C (0.02 C min^–1^), (II) temperature adjustment to 25 °C (10 min), followed by a frequency sweep at 25 °C (γ = 0.01, ω = 100–0.01 rad s^–1^), (III) temperature adjustment to 10 °C (10 min), followed by another three-segment thermal treatment with the same condition as Step I but at a doubled heating and cooling rate (0.04 C min^–1^) and a shorter isothermal segment (5 min), (IV) temperature adjustment to 25 °C (10 min), followed by a step–strain measurement at 25 °C (γ = 0.1), and temperature adjustment to 40 °C (10 min), followed by another step–strain at 40 °C (γ = 0.1), (V) temperature adjustment to 25 °C (10 min), a time-sweep (150 s, γ = 0.01, ω = 10 rad s^–1^), an amplitude-sweep (γ = 0.01–10, ω = 1 rad s^–1^), followed by another immediate time-sweep (logarithmic ramp, 470 s, γ = 0.01, ω = 10 rad s^–1^).

Circular dichroism (CD) spectra were recorded on a JASCO CD-spectrometer model J-815, using 0.5 mM peptide conjugate solutions in 10 mM PBS buffer. Around 300 μL of solution was enough to fill the quartz cell with a thickness of 2 mm. All spectra were corrected by subtraction of the plain buffer (background). All samples were measured in a temperature range of 20 to 50 °C, with 10 °C steps. The temperature was equilibrated at each step for at least 5 min. All spectra were averaged over 3 scans between 190 and 300 nm at the rate of 100 nm min^–1^. Curves were further smoothed by linear interpolation in Matlab.

Transmission electron microscopy was performed on a Tecnai T12 system by FEI equipped with a BioTwin lens and a LaB_6_ cathode operating at 120 kV. A MegaSys 1k × 1k CCD sensor was used to capture images. Samples were prepared at concentrations of 50 μM. Samples were prepared by adsorbing 5 μL of the sample onto a glow-discharged CF300-U copper grid coated with a 3–4 nm carbon layer (Electron Microscopy Sciences, Pennsylvania, USA) for 0.5–1 min. All samples were stained for 1 min using 5 μL of a 2 wt % uranyl acetate solution. Excess liquid was removed with grade 4 filter paper by Whatman GE Healthcare BioSciences.

## 3 Results and Discussion

To promote our understanding of the role of metal ions in the assembly of peptides and their amphiphilic polymer conjugates, we synthesize an ABA peptide–polymer conjugate with two external hydrophobic and pH-switchable peptide sequences connected via a hydrophilic, flexible PEG segment. The peptide segment consists of alternating three Phe and two His amino acids with a hydrophobic spacer given by 6-aminohexanoic acid, as depicted in Figure 1. His is an interesting amino acid for designing stimuli-responsive biomaterials, as the imidazole side chain is deprotonated at pH above 6.0, unlocking the pH-triggered self-assembly into secondary structures.^8,12,42^ Self-assembly under such mildly acidic conditions is important in biomedical contexts, being characteristic of tumor microenvironments and inflammatory sites.^43^ Moreover, deprotonated His groups can associate with metal ions, providing the mechanical toughness in mussel threads,^29^ acting as molecular glue that holds proteins in their functional shape,^44^ and helping heme-containing enzymes to convey oxygen, water, or other small molecules.^45^ Furthermore, Phe is identified as the smallest unit and the core recognition motif of the Alzheimer’s β-amyloid polypeptide.^18,46^ As a hydrophobic unit with bulky side groups it plays a primary role in stabilizing the β-sheet structure in aqueous environments.^3,47^ The π–π stacking of bulky aromatic side groups, assisting β-sheet formation, is reported to be enhanced upon interaction with metal ions.^18,36^ As such, the alternating placement of His and Phe is expected to promote the formation of secondary structures, as illustrated in Figure 1.^48^ Moreover, the selected peptide conjugate is expected to not only show pH-switchable structure formation but also respond to metal ion content and identity.

To investigate the effect of metal ions on the secondary structure of peptide self-assembly, we first study the structure formation in the plain peptide–polymer conjugate solution. At low pH values, the CD spectrum of a 0.15 mM (∼0.07 wt %) solution exhibits a positive band at λ = 220 nm and decreases to negative values below 200 nm, as shown in Figures 2a and S5. No CD bands were identified above 240 nm. Upon increasing the pH, the positive band at 200 nm and the negative ones below 195 nm decrease and a strong positive band emerges and increases at λ = 200 nm.^12,13,34,42,49,50^ We have already shown that the same peptide synthon, in an amphiphilic dendrimer design shows a negative band at λ = 205 nm and a weak positive one at 220 nm, indicative of molecularly dissolved chains.^51,52^ Upon increasing the pH above 6.5 a strong negative band appeared at λ = 215 nm and a weaker band at 255 nm, which we interpreted as β-sheet structures. The pH-switchable assembly occurs by the deprotonation of His groups above the p*K*_a_ value of the imidazole side chain. This reduces charge repulsion and allows the peptide sequences to pack more closely into β-sheets. aMD simulations for the same oligopeptide segments and Ramachandran plots suggested an antiparallel β-sheet conformation between neighboring segments.^50^ In agreement with those previous investigations,^50^ the PEG-peptide conjugates in this study show a polypeptide II (P_II_) structure in the unfolded state at pH = 5.5 and upon increasing the pH, the strong positive band at 200 nm can be associated with β-sheet secondary structure formation. The slight red shift compared to corresponding characteristic bands of the plain peptide sequence can be associated with the less tightly packed β-sheet due to the steric demand of the PEG chains.^12,53,54^

**Figure 2:**
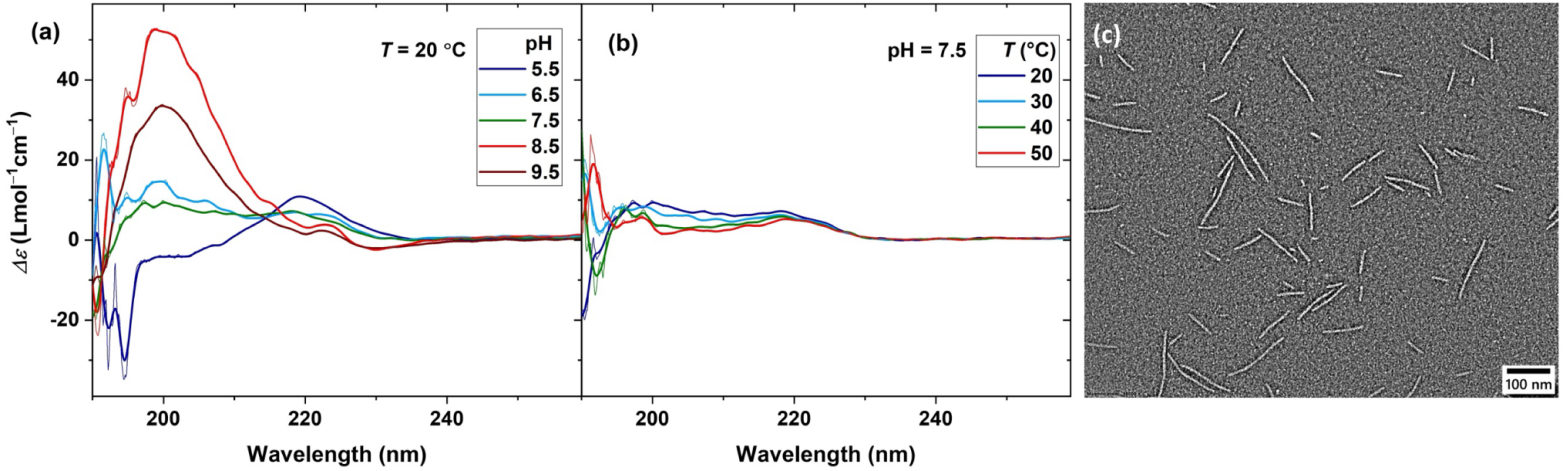
CD spectra of the plain PEG–peptide conjugate as a function of (a) pH and (b) temperature; raw data are overlaid with smoothed data for demonstration. (c) TEM image at pH = 7.5 and room temperature (scale bar = 100 nm).

While the thermo-gelation of the majority of peptide–polymer conjugates is achieved by utilizing thermoresponsive polymer segments, mostly relying on lower-critical solution temperature (LCST) type behavior, the secondary association of peptide segments can also show temperature responsivity.^2,15^ The corresponding effect of temperature on the CD spectrum of the solution at pH = 7.5, as shown in Figure 2b, suggests that secondary structure formation is decreased at higher temperatures. Nanofibrils are not expected to be interconnected at such low concentrations, therefore, the entropic penalty of chain stretching should be negligible.^12,42,55^ Consequently, secondary structures are expected to be driven by the enthalpic gain of physical interactions, such as hydrogen bonding and π–π stacking. Accordingly, the formation of secondary structures decreases, as such interactions are weakened by the temperature. A similar trend is observed at all other pH values, as shown in Figure S5.

To visualize the assembly of peptide sequences in ordered structures, the polymer conjugate solution (50 μM, ∼0.02 wt %) in 20 mM Tris buffer at pH = 2 was neutralized to pH 7.5, drop-cast on carbon-coated copper grids, and stained using uranyl acetate. The uranyl acetate is a negative staining agent: the material of interest scatters less (appears white), and the background is dark.^56^ The obtained TEM images indeed demonstrate the formation of nanofibers, as shown in Figure 2c, with a narrow thickness of 5.7 nm and length averages of *L*_*n*_ = 72 nm and *L*_*w*_ */L*_*n*_ = 1.3, as quantified from the histogram shown in Figure S6. The contour length of a fully extended acetylated pentapeptide sequence extended with an apolar aminohexanoic acid spacer is roughly 3.6 nm, while the radius of gyration of the PEG spacer in the random coil conformation is about 1.5 nm. Combining CD spectroscopic and TEM microscopic data, we hypothesize that the core structure of the nanofibers is based on densely packed β-sheet strands with a unimolecular thickness, which is stabilized in buffer media via a hydrophilic shell of solvated PEG.

At concentrations higher than φ = 1 wt %, increasing the pH above 6 transforms the transparent solution into a turbid and weak hydrogel. To quantify the structure and dynamics of the hydrogel at various pH and temperature values, we performed small-amplitude oscillatory shear measurements. The plateau of the storage modulus reflects the number of active network strands per volume, while the inverse of the frequency at which storage and loss moduli cross (*G*′ = *G*″) is usually considered as the lifetime of transient cross-links.^25,57,58^ The 2 wt % hydrogel demonstrates a flat storage modulus, showing no crossover with the loss modulus, all along the measured frequency range, as shown in Figure S7, which is typical of stable chemically cross-linked networks.^58^ The network structure is expected to form upon the interconnection of nanofibrils with PEG spacers. The coil overlap concentration of a 3 kg mol^–1^ PEG is above 60 g L^–1^,^59^ therefore, in the 2 wt % hydrogel, PEG chains are significantly stretched, imposing an entropic penalty over network formation. This entropy creates network defects, specifically dangling free PEG segments and intrananofibril connections, which form loops. Our attempt to construct a master curve by time–temperature superposition (TTS) was unsuccessful, as *G*′ and *G*″ demonstrated significant vertical shifts at high temperature. Specifically, while the moduli are in the same range at 10 and 25 °C, they show a jump at 40 °C, with the largest increase for hydrogels obtained at higher pH values, as shown in Figure S7c. According to the CD spectroscopy results (Figure 2), β-sheet-driven nanofibrils are destabilized at higher temperature. These nanofibrils form multivalent junctions of the network. Upon increasing the temperature, while the number of network strands (number of PEG chains per volume) is constant, the number and functionality of cross-links (length of nanofibers) decrease. Decreasing the junction functionality is expected to decrease the plateau modulus according to the phantom network model.^23,40,60^ However, junctions of lower valency decrease defects and increase the effective number of interconnections at such concentrations, as network strands become less stretched. Therefore, the counterintuitive increase of modulus at higher temperatures, despite the decreased tendency of peptide sequences to form secondary structures, is due to lower chain stretching.

To better understand the temperature dependency of the network structure and dynamics, we performed a three-step oscillatory thermal treatment including a heating ramp and isothermal time-sweep, followed by a cooling ramp. The obtained results, as shown in Figure 3a for the hydrogel at pH = 6.5, show a significant thermal history-dependent material property. While heating results in smooth network buildup, the modulus continues to grow in the isothermal segment, suggesting that the network buildup by continuous adaptation of nanofibril length is slower than the selected heating rate of 0.02 °C min^–1^. Interestingly, the follow-up cooling segment shows an initial network buildup until 40 °C followed by a nonlinear structure formation. The storage modulus reached at 10 °C is less than half of the starting modulus, indicative of the aging effect on the structure of the starting material. A second three-step thermal treatment, at doubled heating and cooling rates, shows a similar temperature-dependent modulus. This aging effect is obvious in hydrogels obtained at other pH values, as shown in Figure S8, and as reported for other peptides and their polymer conjugates.^47,61^ Interestingly, there is a lag between the network formation during cooling in the first and second runs, which is amplified by the pH value (see Figure S8), implying enhanced stability at higher pH. While we cannot exactly determine the origin of this complex temperature dependency, it shows that the time-dependent network formation significantly accelerates around 40 °C. This also explains the jump in storage modulus at 40 °C, reflected in SAOS data shown in Figure S7. Consequently, to set a basis for comparing the network structure at various pH values, we chose to measure the frequency sweep at 25 °C after the first thermal treatment. The obtained dynamic moduli, as shown in Figure 3b, demonstrate a rather decreasing trend upon an increase in the pH value. This is in line with CD spectroscopy results, suggesting more pronounced secondary structure formation at higher pH values. As noted earlier, the growth of nanofibrils results in higher junction valency and more chain stretching, which creates more defects and less load-bearing network strands.

**Figure 3:**
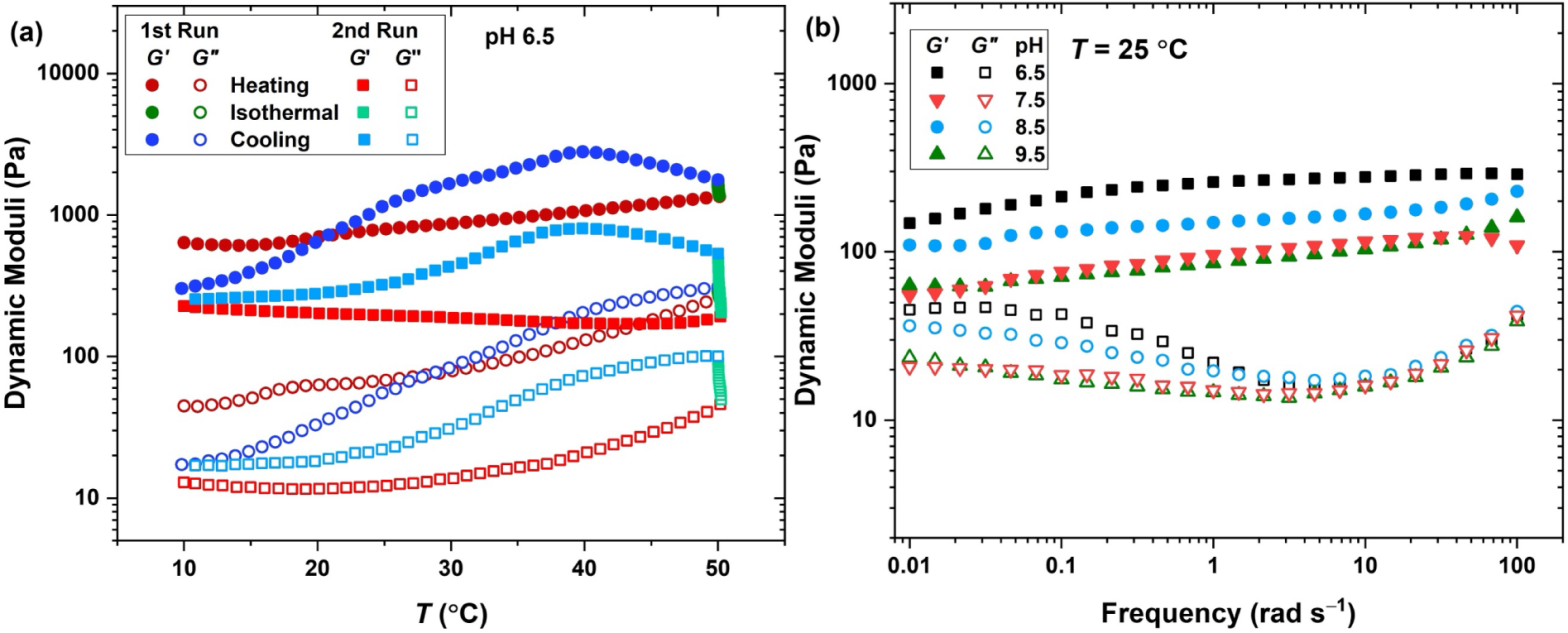
(a) Oscillatory thermal treatment (γ = 1%, ω = 10 rad s^–1^, pH = 6.5) including subsequent heating, isothermal, and cooling segments at rates of 0.02 (1st Run) and 0.04 C min^–1^ (2nd Run). (b) Dynamic storage (filled symbols) and loss (open symbols) moduli at various pH values (φ = 2 wt %).

As the network relaxation time, comprehended from the crossover frequency, is located below the accessible frequency range that could not be extended due to the TTS failure, we performed step–strain measurements at 25 and 40 °C. The obtained results, as represented in Figure 4a for the hydrogel at pH = 7.5, demonstrate that the network relaxation is a very slow and broad process, which could not be captured using a single exponential decay function. Therefore, we fit the relaxation modulus with a generalized Maxwell model consisting of a Gaussian distribution of relaxation times.^25,62^ This gives us an estimate of the network relaxation process. The fit parameters, including the average relaxation time, the standard deviation, and the plateau modulus, as listed in Table S1, demonstrate that all samples have a broad relaxation process, which becomes faster at the higher temperature. The broad relaxation process is another indication for the distribution of the nanofiber length, which is amplified at higher pH values, as shown in Figure S9 for hydrogels at other pH values.

**Figure 4:**
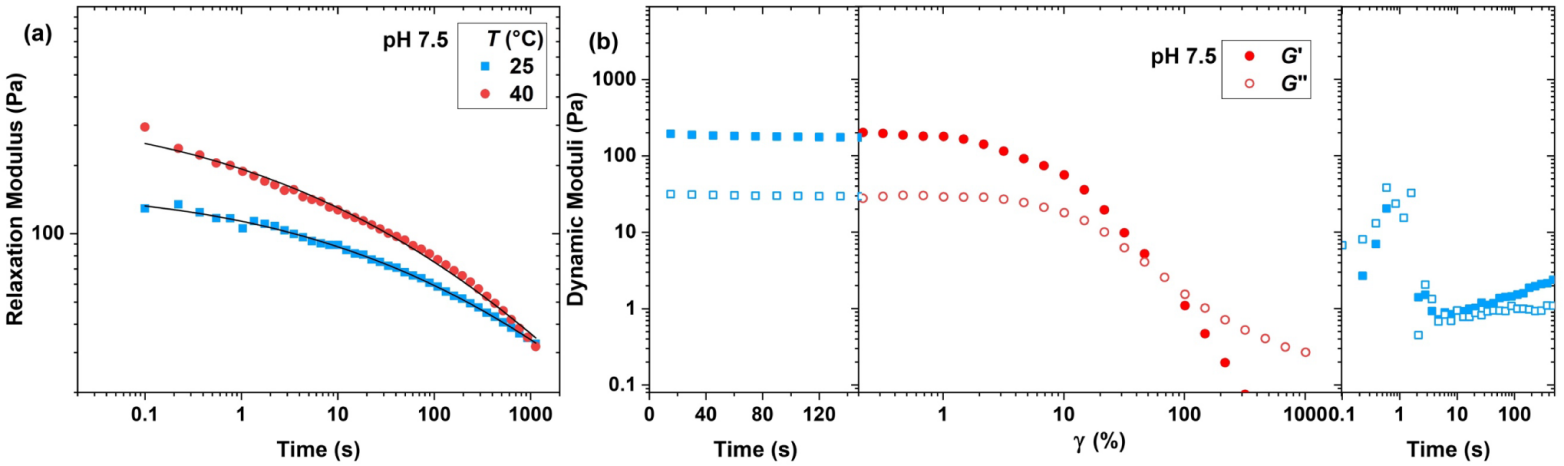
(a) Relaxation modulus at various temperatures (symbols) and the fit of the generalized Maxwell model (lines) (γ = 10%, pH = 7.5, φ = 2 wt %). (b) Dynamic storage (filled symbols) and loss (open symbols) moduli in network destruction and recovery, including time sweeps (γ = 1%, ω = 10 rad s^–1^) before and after an amplitude sweep (ω = 10 rad s^–1^, *T* = 25 °C).

Having a better overview of the network structure, we subsequently studied the network destruction and recovery through an amplitude sweep up to 1000% oscillatory deformation, followed by an immediate time sweep. The obtained results, as shown in Figure 4b, indicate that the onset of a nonlinear viscoelastic region is reached at oscillation amplitudes as low as 5%, and the network recovery after 1000% deformation is very slow. This is in agreement with the slow aggregation of nanofiber cross-links and overstretched PEG strands.

The results obtained so far suggest that although this concentration level is relevant for the biomedical application of peptide conjugates,^12,15,49,63^ the mechanical properties are compromised due to limited accessibility of chain ends for effective cross-linking. To explore this further, we investigated the effects of slight variations in the concentration. The thermal treatment, as represented in Figure 5a for the hydrogel at φ = 3.0 wt % and pH = 7.5, reveals a gradual network buildup during heating and the subsequent isothermal time sweep, followed by a nonlinear network breakdown upon cooling. Interestingly, the lag in network breakdown during cooling between the first and second runs amplifies with concentration, as shown in Figure S10, indicative of the formation of more stable networks at higher concentrations. Furthermore, the storage modulus reached at the end of the experiment stays above the initial value in both runs, for concentrations above 2 wt %, suggesting that new nucleation and fibril growth can occur upon thermal treatment at high concentrations.^64^

**Figure 5:**
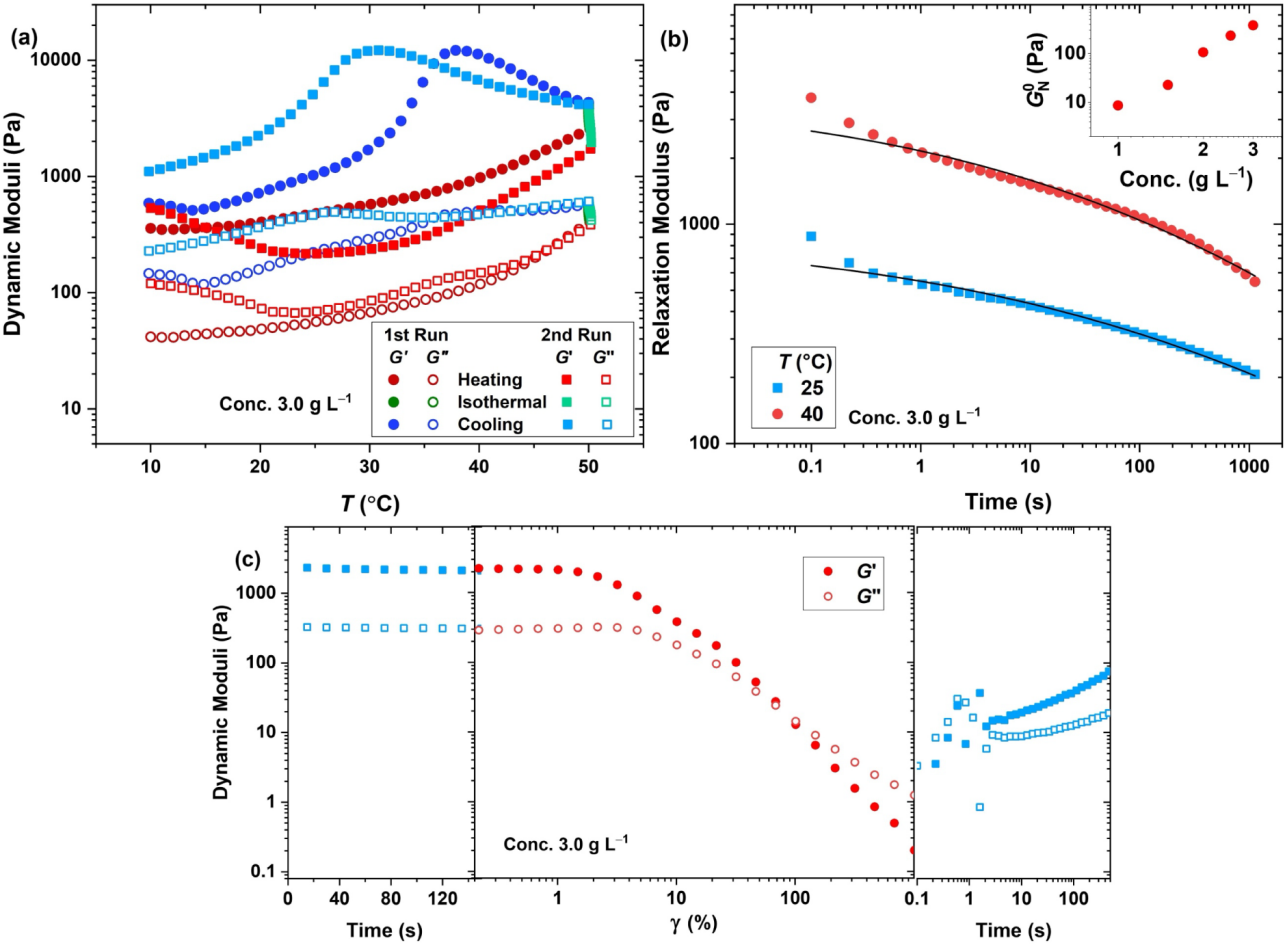
(a) Oscillatory thermal treatment (γ = 1%, ω = 10 rad s^–1^) of the hydrogel obtained at φ = 3.0 wt %. (b) Relaxation modulus at various temperatures (symbols) and the fit of the generalized Maxwell model (lines) (main plot) and the plateau modulus extracted from the frequency sweep (inset plot). (c) Dynamic storage (filled symbols) and loss (open symbols) moduli in network destruction and recovery (ω = 10 rad s^–1^, pH = 7.5, φ = 3.0 wt %, *T* = 25 °C).

Frequency-sweep measurements confirm typical gel-like behavior, with *G*′ staying above *G*″ across the entire frequency range and no observable crossover, as shown in Figure S11. The plateau modulus, compiled and depicted in the inset of Figure 5b, exhibits a power-law dependence on concentration, consistent with a Rouse-like relaxation mechanism expected for nonentangled polymer chains.^58^ The relaxation modulus shows a faster drop at higher temperatures, as shown in the main plot of Figure 5b for the hydrogel at φ = 3.0 wt % and pH = 7.5. Interestingly, the step–strain measurement does not indicate slower relaxations at high concentrations, as shown in Figure S12. The fit parameters used to explain the data by the generalized Maxwell model, as listed in Table S2, suggest that the relaxation time and broadness oscillate with no consistent trend. Similarly, the onset of the nonlinear viscoelastic region remains largely unaffected by concentration, as shown in Figure 5c, while the network recovery becomes faster when the concentration is raised from 2 to 3 wt % (compared to Figure 4b). Overall, these observations indicate that a modest increase in concentration promotes nanofiber formation, consequently enhances network connectivity, and accelerates structure recovery due to increased mutual accessibility of chain ends.

Having this deep insight from the structure and dynamics of networks made by the plain PEG–peptide conjugate, we study the effect of metal ions on the formation of secondary structures. Specifically, we employ Zn^2+^, as it is one of the most abundant transition metals in living organisms and is frequently found bound to His residues in proteins, stabilizing their structure and function,^17,21^ or amplifying the aggregation of amyloid-β peptides.^65^ Accordingly, we first added the metal ion solution to the PEG–peptide solution under acidic conditions and then adjusted the pH to the desired value. Increasing the Zn:His molar ratio from 0 to 2 results in profound changes in the CD spectrum, as shown in Figure 6a. This includes an initial enhancement of the β-sheet characteristic positive band at 200 nm up to a Zn:His ratio of 0.35, followed by a decline of the β-sheet characteristic band and a gradual growth of two negative bands at λ = 203 and 214 nm, as the Zn:His ratio increases, which can be associated with the characteristic bands of an α-helical structure.^1,54^ CD spectra obtained at higher temperatures, as shown in Figures 6b and S13, suggest that while peptide assembly in the form of a β-sheet is reduced by temperature, the formation of α-helices is increased. However, the temperature dependency is lost at the highest Zn^2+^ content, as represented in Figure S13 for the hydrogel formed at Zn:His = 1.00. Evidently, Zn^2+^ coordinates to His imidazole N atoms, creating specific His–Zn–His bridges at pH > 6.5. At low stoichiometry (<0.5 equiv), Zn^2+^ preferentially cross-links subsets of His residues, improving the order and stability of β-sheets. At higher stoichiometry, the monocomplexation of imidazole ligands seemingly disrupts the transient cross-linking of β-sheets, instead supporting the formation of α-helical domains up to a ratio of Zn:His = 1.00. These have higher temperature stability, as reflected in reduced thermal fluctuations.

**Figure 6:**
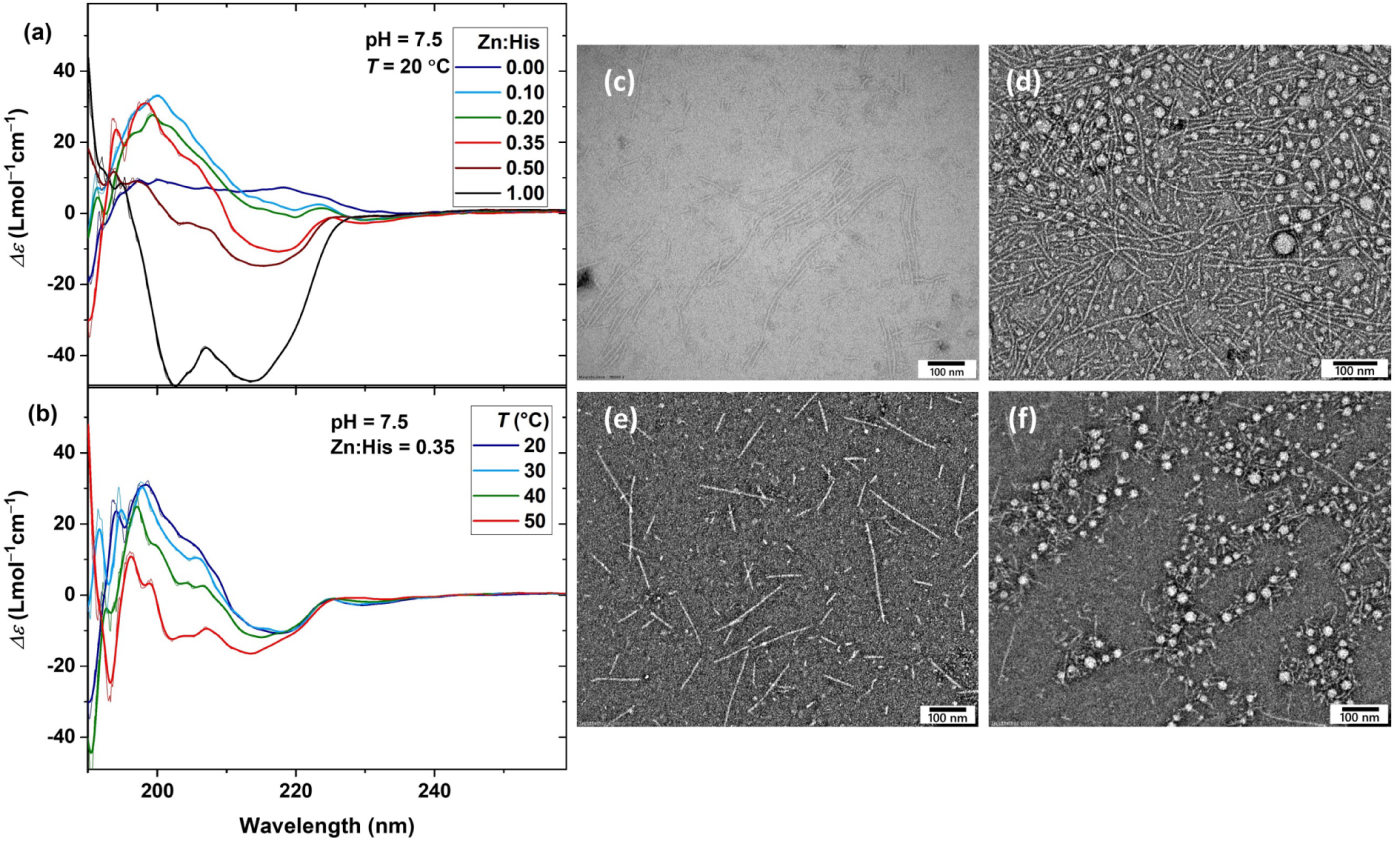
CD spectra of the PEG–peptide solution as a function of (a) Zn:His ratio and (b) temperature (φ = 0.5 mM, pH = 7.5); raw data are overlaid with smoothed data for demonstration. TEM images at pH = 7.5 and room temperature obtained at Zn:His ratios of (c) 0.1, (d) 0.2, (e) 0.5, and (f) 1.0 (scale bar = 100 nm).

The corresponding TEM images demonstrate the formation of long nanofibers, along with the parallel appearance of spherical nanoparticles at Zn:His of 0.2 and 1.0, as shown in Figure 6c–f. The histogram of nanofiber lengths, as shown in Figure S14 and compiled in Figure S15, indicates that the number-average nanofiber length roughly doubles upon the introduction of Zn^2+^ up to the Zn:His ratio of 0.2, and then gradually decreases at higher Zn^2+^ concentrations. In contrast, the nanofiber thickness remains essentially unchanged in the presence of Zn^2+^, varying only slightly between 6 and 6.8 nm across different samples. The spherical nanoparticles also exhibit a consistent number-average diameter of approximately 20 nm. These spherical nanoparticles are frequently associated with disordered peptide aggregates, specifically in the presence of Zn^2+^.^1,26,34,54^ The combination of CD spectra and TEM images suggests that Zn^2+^ does not change the lateral bundling of nanofibers; however, through improved packing of β-sheets and their transient bonding, it can promote their growth in length. The loss of temperature sensitivity of the CD spectra suggests that Zn^2+^ further locks the secondary structures. The formation of bis-His complexes^29,30^ and promoted π–π stacking of aromatic side chains in Phe groups contribute to this structure formation.^18,36^

The metal ion-induced modulation of the secondary structure is expected to have a direct effect on the structure and stability of the obtained hydrogels. The addition of the metal ion did not change the viscosity, indicating that complexation with His or promotion of stacking of Phe side chains is not possible under acidic conditions. Nevertheless, it evidently has an impact on the hydrogel properties at pH > 6.5. Indeed, the three-step thermal treatment, as shown in Figure 7a, reveals new features in the presence of Zn^2+^. For the hydrogel formed at Zn:His = 0.2 (φ = 2 wt % and pH = 7.5), the heating-induced network buildup and the cooling-promoted breakdown proceed more gradually and steadily compared to the metal-free network. The higher stability of multivalent junctions is translated into a less modulus increase upon heating and reduced lag between the network breakdown upon cooling in the first and second runs, as also witnessed for hydrogels formed at other Zn:His ratios, shown in Figure S16. Moreover, the aging effect is minimized as the initial and final storage moduli are in the same range, all indicative of a more dynamic structure in the presence of Zn^2+^. The dynamic moduli obtained by frequency sweep, as shown in Figure S17, demonstrate the same gel-like behavior with no indication of crossover. Also, there is no sign of an early relaxation mode associated with bis-Zn^2+^–His complexes, as usually seen in model dual-network hydrogels.^66^ This is reasonable as metal complexes do not make network cross-links; instead, they contribute to the stability and functionality of nanofibril junctions. The corresponding plateau modulus, as compiled in the inset plot of Figure 7b, follows the same trend as it was seen for the average length of nanofibers, shown in Figure S15. This suggests that, in this range of nanofibril length, the effect of junction functionality in network connectivity outweighs the chain stretching penalty; therefore, it directly correlates with the modulus. Accordingly, two times increase in the length of nanofibers upon introducing 0.2 equiv Zn^2+^ translates into more than one order-of-magnitude increase in the plateau modulus.

**Figure 7:**
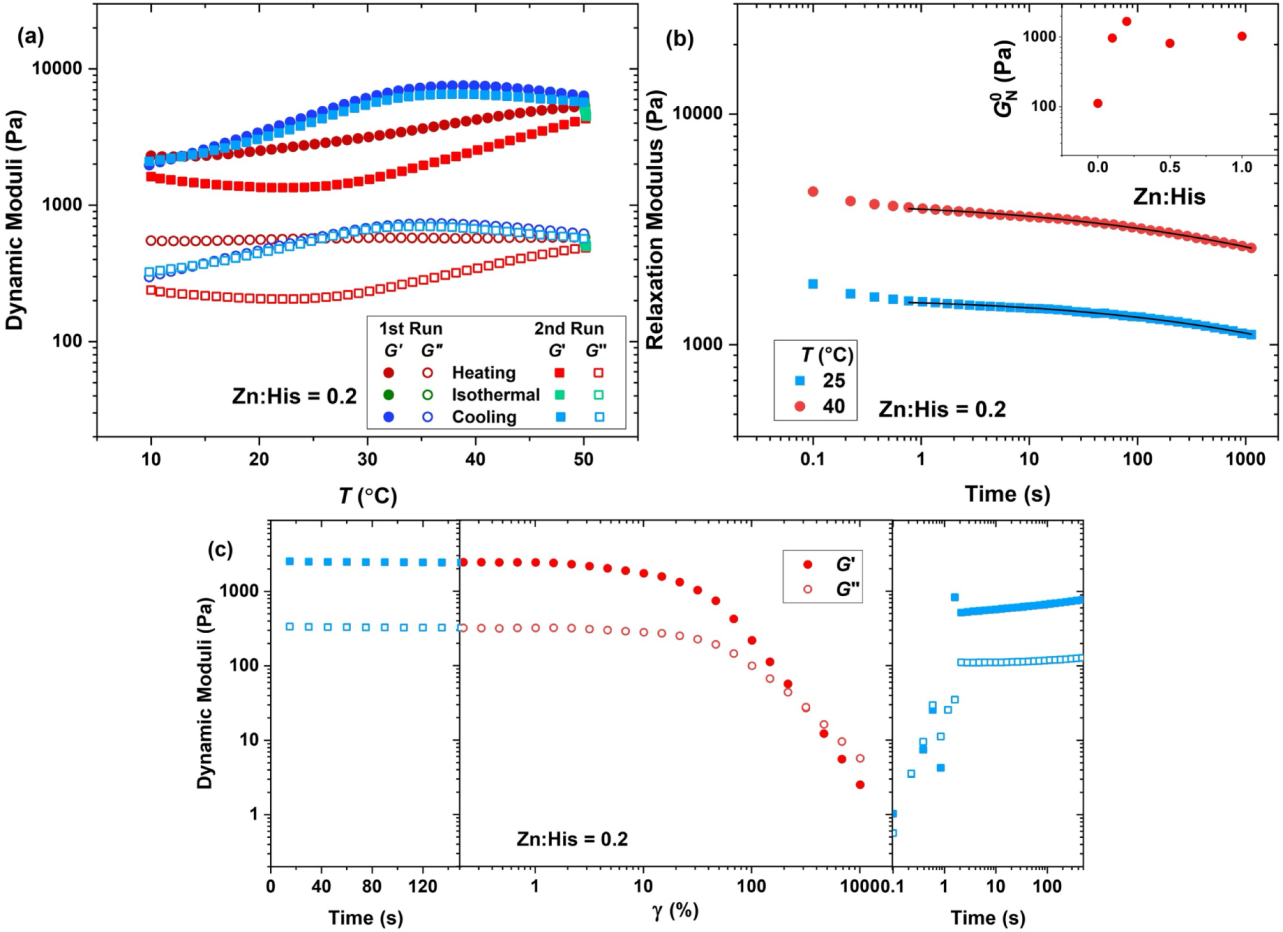
(a) Oscillatory thermal treatment (γ = 1%, ω = 10 rad s^–1^, pH = 7.5) of the hydrogel obtained at Zn:His = 0.2. (b) Relaxation modulus at various temperatures (symbols) and the fit of the generalized Maxwell model (lines) (main plot) and the plateau modulus extracted from the frequency sweep (inset plot). (c) Dynamic storage (filled symbols) and loss (open symbols) moduli in network destruction and recovery (ω = 10 rad s^–1^, pH = 7.5, φ = 2.0 wt %, Zn:His = 0.2, *T* = 25 °C).

To better assess the stability of nanofibers, we performed step–strain measurement, as represented for the hydrogel at Zn:His = 0.2 in the main plot of Figure 7b. The fit parameters of the generalized Maxwell model, as listed in Table S3, indicate that the network relaxation time increases monotonically with Zn^2+^ content, demonstrating that not only bridging His ligands with Zn^2+^ ions, but also the formation of monocomplexes contribute to the stability of secondary structures, as shown in Figure S18 for hydrogels obtained at other Zn:His ratios. Interestingly, Zn^2+^ also shifts the onset of the nonlinear viscoelastic region to higher deformations, as represented for the hydrogel at Zn:His = 0.2 in Figure 7c, and for other Zn:His ratios in Figure S19. Furthermore, although the hydrogels cannot be considered self-healing, the recovery rate after network breakdown is significantly enhanced in the presence of Zn^2+^ ions, as shown in Figure 7c. This effect clearly arises from the rapid reformation of Zn^2+^–His coordinative bonds, the process that is reported to be responsible for the self-healing and toughness of mussel threads.^21,28,34^

The addition of Zn^2+^ appeared promising, enhancing the secondary structure formation and the consequent network mechanics. Given the complex influence of coordination geometry on the peptide assembly, we further investigated the effect of adding other metal ions with distinct geometries. For this purpose, Cu^2+^, Ni^2+^, and Co^2+^ were selected, as they are more frequently studied in complexation with His.^18,21,27,31,37,67^ While copper and cobalt ions are frequently used in redox-responsive systems,^67,68^ there is no risk of oxidation in this study. Cu^2+^ is quite stable in water solution, and Co^2+^ can turn into Co^3+^ only in the presence of a strong oxidant.^67^ As longer nanofibers and enhanced network mechanics were observed at Zn:His = 0.2, this metal content is the focus of subsequent experiments.

Introducing other metal ions into the PEG–peptide assembly has quite similar effects on the CD spectrum, as shown in Figure 8a, which is surprisingly different from the effects of Zn^2+^. A better overview of the effect of temperature and M^2+^:His ratio for different metal ions is provided in Figure S20. Accordingly, at low metal ion contents, all metal ions enhance the intensity of the β-sheet characteristic bands, even more than Zn^2+^. However, in contrast to Zn^2+^, which seems to stabilize α-helices at higher concentrations, other metal ions keep the same signatures of the β-sheet structure with slightly lower intensities. In addition, unlike Zn^2+^, the situation does not change upon increasing the temperature; the β-sheet characteristic bands persist but decrease in intensity at higher temperatures. This discrepancy can be associated with the interplay of metal–imidazole coordination geometry of the histidine side chain and secondary structure formation of the oligopeptide backbone.^9,26,28,34^ Presumably, the tetrahedral geometry preference of Zn^2+^ should be less compatible with the planar assembly of β-sheets, necessitating a twist in the organization of the assemblies. In contrast, the other ions can better adjust to the planar assembly, due to the flexibility of their preferred octahedral geometry, which can be fulfilled by the inclusion of small molecules, like solvent and counterion, in case of steric strains.

**Figure 8:**
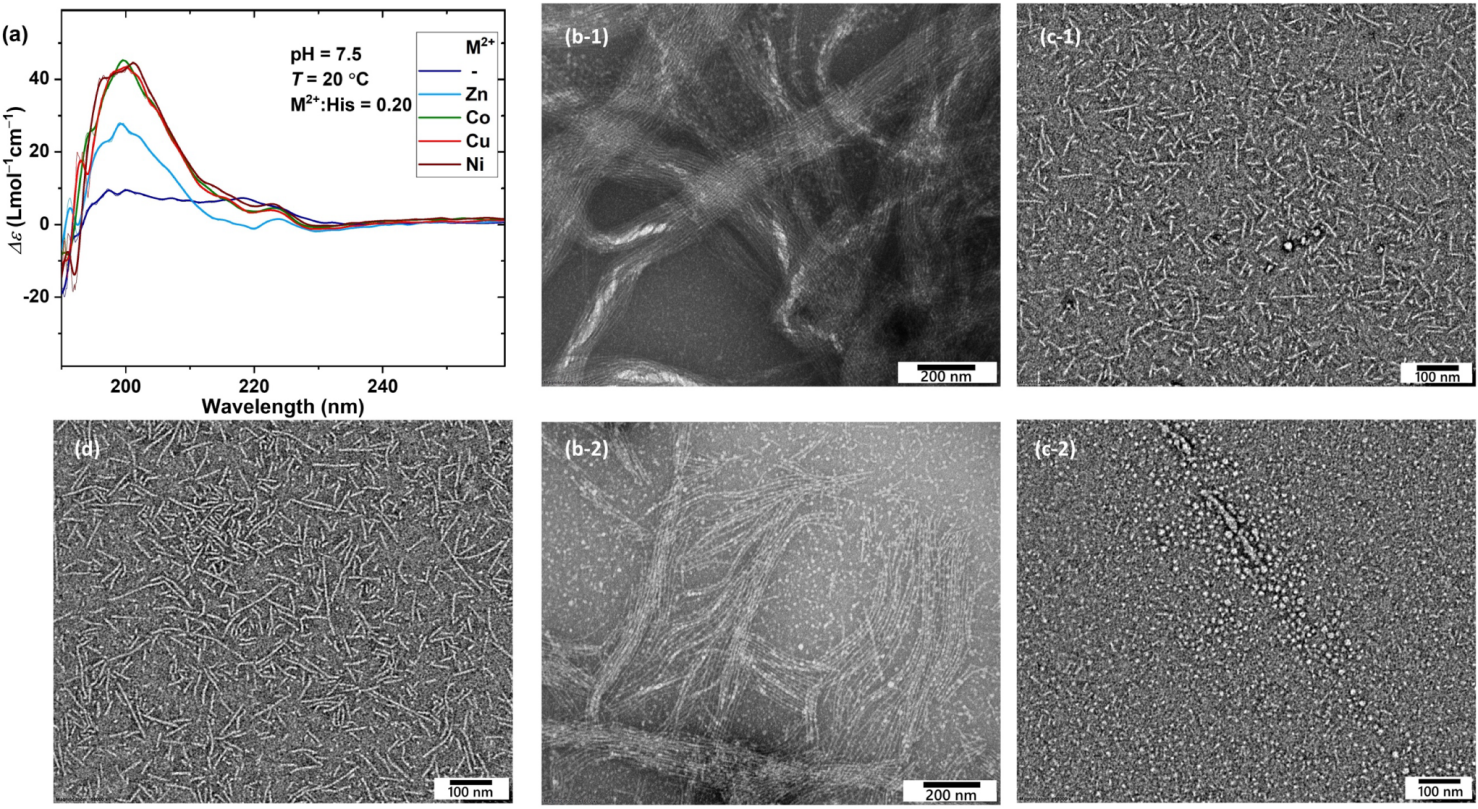
(a) CD spectra of the PEG–peptide hydrogels obtained in the presence of different metal ions at M:His = 0.2; raw data are overlaid with smoothed data for demonstration. TEM images at pH = 7.5 and room temperature obtained in the presence of different metal ions: (b) Co^2+^ (scale bar = 200 nm), (c) Cu^2+^ (scale bar = 100 nm), and (d) Ni^2+^ (scale bar = 100 nm).

To corroborate findings from CD spectroscopy, we performed further TEM investigations. The corresponding TEM images demonstrate the formation of extremely long nanofibers, in the order of micrometer in length and 7.0 nm in thickness, upon the introduction of Co^2+^ at Co:His = 0.2, as shown in Figure 8b. Some small spherical nanoparticles with a number-average diameter of 9.8 nm could also be observed, as highlighted in Figure 8b-2 and quantified in Figure S21. The introduction of Cu^2+^ results in the largest fraction of spherical nanoparticles (*D_n_* = 6.2 nm, *D_w_*/*D_n_* = 1.12) along with nanofibrils, as shown in Figure 8c. With a number-average length of 46.3 nm and thickness of 6.0 nm, these nanofibers are half in length compared to those obtained in the plain PEG–peptide samples. In contrast, Ni^2+^ promotes the pure formation of nanofibers with a number-average length of *L*_*n*_ = 56.2 nm and *L*_*w*_ */L*_*n*_ = 1.3, and thickness of 7.0, as shown in Figure 8d and quantified in Figure S21.

Accordingly, CD is sensitive to local secondary structure and not global assembly dimensions. The different nanofiber lengths formed in the presence of the latter metal ions despite their similar CD spectra suggest that they can stabilize a similar β-sheet conformation but follow different kinetics of assembly. Different nanofiber lengths presumably arise from ion-dependent coordination kinetics besides the nucleation and growth behavior of the peptide segments, which affect the assembly order without alteration of the underlying β-sheet conformation.

FTIR provides further insightful information about the oligopeptide assemblies and can support conclusions drawn from CD spectroscopy analysis. Accordingly, we performed FTIR measurements of the 2 wt % hydrogels, as shown in Figure S22. FTIR spectra show an amide I band in between 1630–1640 cm^–1^ for all samples, which is a classic signature of β-sheet hydrogen bonding in peptides.^27,70^ This band intensifies and broadens in the presence of metal ions, indicating stronger hydrogen bonding interactions. In the amide II region, between 1500 and 1600 cm^–1^, Zn^2+^, Cu^2+^, and Ni^2+^ show slightly higher intensities compared to Co^2+^, suggesting stronger hydrogen bonding and amide interactions.

The hydrogel obtained in the presence of Co^2+^ had a blue hue, as shown in Figure 9a, in contrast to those produced with other metal ions, which had a colorless turbid appearance. This photochemical properties of Co^2+^–His complexes have made them useful as a rapid and reliable sensor for oxygen detection.^71^ The extraordinary longitudinal growth of nanofibers in the presence of Co^2+^ is expected to have a profound effect on the mechanical properties of the hydrogels formed at φ = 2 wt % and pH = 7.5. The rheological behavior, however, does not reflect the expected long-fiber-induced jump in properties. Specifically, the three-step thermal treatment shows a structure buildup after heating, which does not tend to breakdown upon cooling, as shown in Figure 9a, so that the modulus reached at the end of the experiment stays an order of magnitude above the starting modulus. This opposite aging effect suggests that fiber length reaches equilibrium over time, balancing junction functionality and chain stretching, thereby minimizing connectivity defects. A similar trend is obtained in the presence of Cu^2+^ and Ni^2+^, as shown in Figure S23. The frequency sweep data, as shown in Figure S24, demonstrate a gel-like behavior in the whole frequency range with no sign of crossover. However, the plateau modulus, as compiled and shown in the inset plot of Figure 9b, significantly drops in the presence of Cu^2+^, while it shows an increase upon the introduction of Co^2+^ and Ni^2+^, but not to the extent that was observed in the presence of Zn^2+^. Accordingly, the correlation of the nanofiber length with plateau modulus that was observed at different Zn^2+^ contents does not hold when comparing different metal ions. With similar fiber lengths and CD spectra, Cu^2+^ and Ni^2+^ demonstrate significantly different modulus levels. A possible explanation for this observation could be the large fraction of less-ordered spherical nanoparticles in the presence of Cu^2+^.

**Figure 9:**
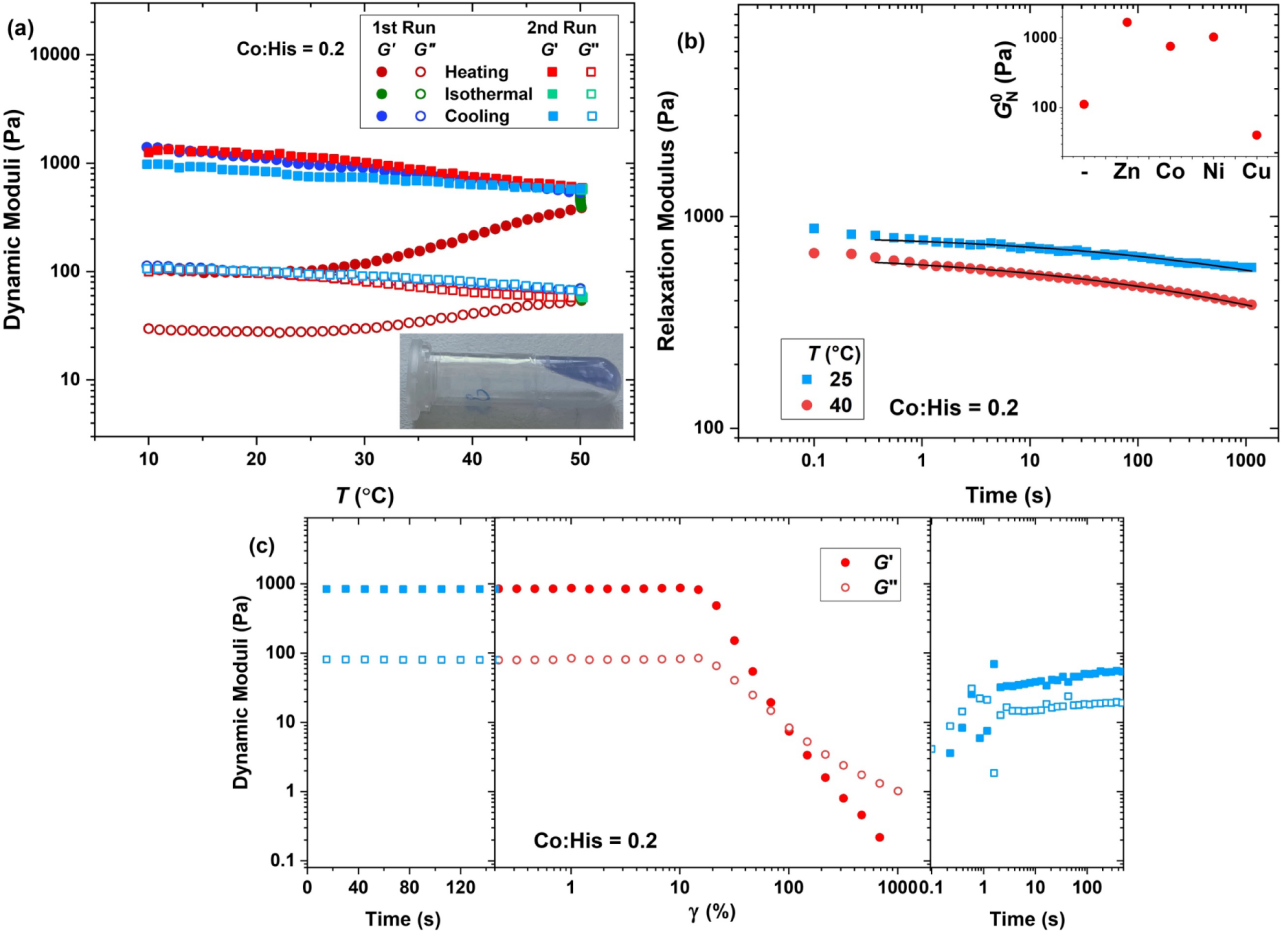
(a) Oscillatory thermal treatment (γ = 1%, ω = 10 rad s^–1^, pH = 7.5) of the hydrogel obtained at Co:His = 0.2. (b) Relaxation modulus at various temperatures (symbols) and the fit of the generalized Maxwell model (lines) (main plot) and the plateau modulus extracted from the frequency sweep (inset plot). (c) Dynamic storage (filled symbols) and loss (open symbols) moduli in network destruction and recovery, including time sweeps (γ = 1%, ω = 10 rad s^–1^) before and after an amplitude sweep (ω = 10 rad s^–1^) (pH = 7.5, φ = 2.0 wt %, Co:His = 0.2, *T* = 25 °C).

The relaxation modulus obtained from the step–strain measurement, as shown in Figures 9b and S21 and quantified in Table S4, reveals that regardless of the plateau modulus level, the relaxation process becomes significantly broader and slower in the presence of metal ions compared to the plain network. Similar to Zn^2+^, all metal ions shift the onset of the nonlinear viscoelastic region to higher deformations and improve the rate of network recovery after breakdown. This is a direct indication for the formation of reversible metal coordination complexes. Nevertheless, Zn^2+^ seems to have the largest contribution to the self-healing properties among the studied metal ions.

## 4 Conclusions

The PEG–peptide conjugate with alternating Phe and His residues demonstrates a pH-induced, enthalpy-driven formation of β-sheets and nanofibril assemblies at low concentrations. However, at high concentrations, they form a percolated network with nanofibril junctions, interconnected by PEG strands. The network connectivity is set by the traditional trade-off between the enthalpic gain of assembly and the entropic penalty of chain stretching. Metal ions strongly modulate the supramolecular organization and mechanical behavior of PEG–peptide hydrogels, altering not only the local packing of secondary structures but also their global assembly. Specifically, Zn^2+^ with its dominant tetrahedral coordination geometry preference initially enhances β-sheet packing and nanofiber length, translated in enhanced storage modulus, but imposes a structural twist at high concentrations, resulting in a loss of network connectivity and modulus. In contrast, Cu^2+^, Co^2+^, and Ni^2+^ enhance the β-sheet packing, nevertheless, this does not result in similar global assemblies, as Co^2+^ gives rise to micrometer-long nanofibers, while the other two metal ions do not significantly change the nanofiber length. Moreover, the nanofiber length does not directly contribute to the network connectivity, as Cu^2+^ significantly reduces the modulus, while the other two metal ions increase the modulus. A common observation is that all metal ions similarly enhance the stability of nanofibers, expand the linear viscoelastic domain, and accelerate network recovery, which originates from the fast association of metal complexes. Overall, these results demonstrate that metal–histidine coordination serves as a versatile handle to tune both the static stiffness and dynamic responsiveness of multidomain peptide-based hydrogels, enabling the rational design of stimuli-responsive soft materials with tailored mechanics.

## Supplementary Material



## Notes

Details of synthesis and characterization, additional spectroscopic data, additional rheological data, analysis of TEM images, and rheological results (PDF)

## References

[1] Housmans J. A., Wu G., Schymkowitz J., Rousseau F. (2023). A guide to studying protein aggregation. FEBS J..

[2] Li Y., Yang G., Gerstweiler L., Thang S. H., Zhao C.-X. (2023). Design
of stimuli-responsive peptides and proteins. Adv. Funct. Mater..

[3] Bonduelle C. (2018). Secondary
structures of synthetic polypeptide polymers. Polym. Chem..

[4] Juković M., Ratkaj I., Kalafatovic D., Bradshaw N. J. (2024). Amyloids, amorphous
aggregates and assemblies of peptides–Assessing aggregation. Biophys. Chem..

[5] Lehn J. M. (2013). Perspectives
in chemistry—steps towards complex matter. Angew. Chem., Int. Ed..

[6] Palomo J. M. (2014). Solid-phase peptide synthesis: an
overview focused
on the preparation of biologically relevant peptides. RSC Adv..

[7] De
Maeseneer T., Cauwenbergh T., Gardiner J., White J. F., Thielemans W., Martin C., Moldenaers P., Ballet S., Cardinaels R. (2024). Peptide Sequence Variations Govern
Hydrogel Stiffness: Insights from a Multi-Scale Structural Analysis
of H-FQFQFK-NH2 Peptide Derivatives. Macromol.
Biosci..

[8] Kemper B., Zengerling L., Spitzer D., Otter R., Bauer T., Besenius P. (2018). Kinetically
controlled stepwise self-assembly of AuI-metallopeptides
in water. J. Am. Chem. Soc..

[9] Knight A. S., Larsson J., Ren J. M., Bou Zerdan R., Seguin S., Vrahas R., Liu J., Ren G., Hawker C. J. (2018). Control of amphiphile self-assembly via bioinspired
metal ion coordination. J. Am. Chem. Soc..

[10] Dai M., Goudounet G., Zhao H., Garbay B., Garanger E., Pecastaings G., Schultze X., Lecommandoux S. (2021). Thermosensitive
hybrid elastin-like polypeptide-based ABC triblock hydrogel. Macromolecules.

[11] Asokan-Sheeja H., Awad K., Xu J., Le M., Nguyen J. N., Nguyen N., Nguyen T. P., Nguyen K. T., Hong Y., Varanasi V. G. (2024). In Situ Synthesis and
Self-Assembly of Peptide–PEG Conjugates: A Facile Method for
the Construction of Fibrous Hydrogels. Biomacromolecules.

[12] Otter R., Henke N. A., Berac C., Bauer T., Barz M., Seiffert S., Besenius P. (2018). Secondary Structure-Driven
Hydrogelation
Using Foldable Telechelic Polymer–Peptide Conjugates. Macromol. Rapid Commun..

[13] Edwards-Gayle C. J., Greco F., Hamley I. W., Rambo R. P., Reza M., Ruokolainen J., Skoulas D., Iatrou H. (2018). Self-Assembly of telechelic
tyrosine end-capped PEO star polymers in aqueous solution. Biomacromolecules.

[14] Hou M., Liu S. (2024). Recent progress of
pH-responsive
peptides, polypeptides, and their supramolecular assemblies for biomedical
applications. Biomacromolecules.

[15] Pascouau C., Schweitzer M., Besenius P. (2024). Supramolecular Assembly and Thermogelation
Strategies Using Peptide–Polymer Conjugates. Biomacromolecules.

[16] Khare E., Holten-Andersen N., Buehler M. J. (2021). Transition-metal
coordinate bonds for bioinspired macromolecules with tunable mechanical
properties. Nat. Rev. Mater..

[17] Holten-Andersen N., Jaishankar A., Harrington M. J., Fullenkamp D. E., DiMarco G., He L., McKinley G. H., Messersmith P. B., Lee K. Y. C. (2014). Metal-coordination:
using one of nature’s tricks
to control soft material mechanics. J. Mater.
Chem. B.

[18] Ji W., Yuan C., Zilberzwige-Tal S., Xing R., Chakraborty P., Tao K., Gilead S., Yan X., Gazit E. (2019). Metal-ion modulated
structural transformation of amyloid-like dipeptide supramolecular
self-assembly. ACS Nano.

[19] Wu B., Hanay S. B., Kimmins S. D., Cryan S.-A., Hermida Merino D., Heise A. (2022). Ion-triggered hydrogels
self-assembled
from statistical copolypeptides. ACS Macro Lett..

[20] Sharma P., Kaur H., Roy S. (2019). Inducing differential
self-assembling
behavior in ultrashort peptide hydrogelators using simple metal salts. Biomacromolecules.

[21] Schmidt S., Reinecke A., Wojcik F., Pussak D., Hartmann L., Harrington M. J. (2014). Metal-mediated
molecular self-healing in histidine-rich
mussel peptides. Biomacromolecules.

[22] Wang L., Yin Y.-L., Liu X.-Z., Shen P., Zheng Y.-G., Lan X.-R., Lu C.-B., Wang J.-Z. (2020). Current
understanding of metal ions in the pathogenesis of Alzheimer’s
disease. Transl. Neurodegener..

[23] Ahmadi M., Yazdanimoghaddam R., Sharif F. (2023). The network structure
in transient
telechelic polymer networks: extension of the Miller–Macosko
model. Phys. Chem. Chem. Phys..

[24] Hosseinzadeh B., Ahmadi M. (2022). Coordination
geometry in metallo-supramolecular polymer
networks. Coord. Chem. Rev..

[25] Ahmadi M., Seiffert S. (2021). Coordination geometry preference
regulates the structure
and dynamics of metallo-supramolecular polymer networks. Macromolecules.

[26] Tunn I., de Léon A. S., Blank K. G., Harrington M. J. (2018). Tuning
coiled coil stability with histidine-metal coordination. Nanoscale.

[27] Jehle F., Fratzl P., Harrington M. J. (2018). Metal-tunable
self-assembly of hierarchical
structure in mussel-inspired peptide films. ACS Nano.

[28] Trapaidze A., D’Antuono M., Fratzl P., Harrington M. J. (2018). Exploring
mussel byssus fabrication with peptide-polymer hybrids: Role of pH
and metal coordination in self-assembly and mechanics of histidine-rich
domains. Eur. Polym. J..

[29] Fullenkamp D. E., He L., Barrett D. G., Burghardt W. R., Messersmith P. B. (2013). Mussel-inspired
histidine-based transient network metal coordination hydrogels. Macromolecules.

[30] Grindy S. C., Lenz M., Holten-Andersen N. (2016). Engineering
elasticity and relaxation time in metal-coordinate cross-linked hydrogels. Macromolecules.

[31] Reinecke A., Brezesinski G., Harrington M. J. (2017). pH-Responsive Self-Organization of
Metal-Binding Protein Motifs from Biomolecular Junctions in Mussel
Byssus. Adv. Mater. Interfaces.

[32] Rammal M., Li C., Reeves J., Moraes C., Harrington M. J. (2023). pH-responsive
reversible granular hydrogels based on metal-binding mussel-inspired
peptides. ACS Appl. Mater. Interfaces.

[33] Danmark S., Aronsson C., Aili D. (2016). Tailoring supramolecular peptide–poly
(ethylene glycol) hydrogels by coiled coil self-assembly and self-sorting. Biomacromolecules.

[34] Tunn I., Harrington M. J., Blank K. G. (2019). Bioinspired histidine–Zn2+
coordination for tuning the mechanical properties of self-healing
coiled coil cross-linked hydrogels. Biomimetics.

[35] Shao T., Noroozifar M., Kraatz H.-B. (2024). Divalent metal ion
modulation of a simple peptide-based hydrogel: self-assembly and viscoelastic
properties. Soft Matter.

[36] Fu W., Sabet Z. F., Liu J., You M., Zhou H., Wang Y., Gao Y., Li J., Ma X., Chen C. (2020). Metal ions modulation of the self-assembly
of short peptide conjugated
nonsteroidal anti-inflammatory drugs (NSAIDs). Nanoscale.

[37] Boyle A. L., Rabe M., Crone N. S., Rhys G. G., Soler N., Voskamp P., Pannu N. S., Kros A. (2019). Selective coordination
of three transition metal ions within a coiled-coil peptide scaffold. Chem. Sci..

[38] Gupta M. K., Becknell K. A., Crosby M. G., Bedford N. M., Wright J., Dennis P. B., Naik R. R. (2018). Programmable
mechanical properties
from a worm jaw-derived biopolymer through hierarchical ion exposure. ACS Appl. Mater. Interfaces.

[39] Ahmadi M., Pareras G., Yeamin M. B., Amann-Winkel K., Rimola A., Poater A., Seiffert S. (2024). Coordination
geometry
and mineralization in self-healing mussel-inspired hydrogels. Chem. Mater..

[40] Ahmadi M., Bauer M., Berg J., Seiffert S. (2024). Nonuniversal Dynamics
of Hyperbranched Metallo-Supramolecular Polymer Networks by the Spontaneous
Formation of Nanoparticles. ACS Nano.

[41] Knecht S., Ricklin D., Eberle A. N., Ernst B. (2009). Oligohis-tags:
Mechanisms of binding to Ni2+-NTA surfaces. J. Mol. Recognit..

[42] Otter R., Klinker K., Spitzer D., Schinnerer M., Barz M., Besenius P. (2018). Folding induced supramolecular assembly
into pH-responsive nanorods with a protein repellent shell. Chem. Commun..

[43] Casey J. R., Grinstein S., Orlowski J. (2010). Sensors and regulators of intracellular
pH. Nat. Rev. Mol. Cell Biol..

[44] López-Laguna H., Sánchez J., Unzueta U., Mangues R., Vázquez E., Villaverde A. (2020). Divalent cations: a molecular glue for protein materials. Trends Biochem. Sci..

[45] Birukou I., Schweers R. L., Olson J. S. (2010). Distal
histidine stabilizes bound
O2 and acts as a gate for ligand entry in both subunits of adult human
hemoglobin. J. Biol. Chem..

[46] Görbitz C. H. (2006). The structure
of nanotubes formed by diphenylalanine, the core recognition motif
of Alzheimer’s β-amyloid polypeptide. Chem. Commun..

[47] Lin Z., Ding J., Chen X., He C. (2023). pH- and Temperature-responsive
Hydrogels Based on Tertiary Amine-modified Polypeptides for Stimuli-responsive
Drug Delivery. Chem. Asian J..

[48] Kumar V. A., Wang B. K., Kanahara S. M. (2016). Rational
design
of fiber forming supramolecular structures. Exp. Biol. Med..

[49] Otter R., Berac C. M., Seiffert S., Besenius P. (2019). Tuning the life-time
of supramolecular hydrogels using ROS-responsive telechelic peptide-polymer
conjugates. Eur. Polym. J..

[50] Obenauer M. L., Dresel J. A., Schweitzer M., Besenius P., Schmid F. (2024). Atomistic
Molecular Dynamics Simulations of ABA-Type Polymer Peptide Conjugates:
Insights into Supramolecular Structures and their Circular Dichroism
Spectra. Macromol. Rapid Commun..

[51] Ahlers P., Frisch H., Holm R., Spitzer D., Barz M., Besenius P. (2017). Tuning the pH-Switch of Supramolecular
Polymer Carriers
for siRNA to Physiologically Relevant pH. Macromol.
Biosci..

[52] Ahlers P., Frisch H., Besenius P. (2015). Tuneable pH-regulated
supramolecular
copolymerisation by mixing mismatched dendritic peptide comonomers. Polym. Chem..

[53] Kelly S. M., Price N. C. (2000). The use of circular
dichroism in the investigation
of protein structure and function. Curr. Protein
Pept. Sci..

[54] Kardos J., Nyiri M. P., Moussong É., Wien F., Molnár T., Murvai N., Tóth V., Vadászi H., Kun J., Jamme F. (2025). Guide
to the structural characterization of
protein aggregates and amyloid fibrils by CD spectroscopy. Protein Sci..

[55] Otter R., Besenius P. (2019). Supramolecular assembly of functional peptide–polymer
conjugates. Org. Biomol. Chem..

[56] Ohi M., Li Y., Cheng Y., Walz T. (2004). Negative staining and image classification—powerful
tools in modern electron microscopy. Biol. Proced.
Online.

[57] Ahmadi M., Nicolella P., Seiffert S. (2022). Network percolation in transient
polymer networks with temporal hierarchy of energy dissipation. Macromolecules.

[58] Rubinstein, M. ; Colby, R. H. Polymer physics; Oxford university press, 2003.

[59] Schmolke W., Ahmadi M., Seiffert S. (2019). Enhancement of metallo-supramolecular
dissociation kinetics in telechelic terpyridine-capped poly (ethylene
glycol) assemblies in the semi-dilute regime. Phys. Chem. Chem. Phys..

[60] Ahmadi M., Jangizehi A., Seiffert S. (2025). Macromolecular Engineering of Self-Healing
in Transient Metallosupramolecular Polymer Networks. Macromolecules.

[61] Yin B., Wang R., Guo Y., Li L., Hu X. (2024). Injectable Thermo-Responsive Peptide Hydrogels and
Its Enzyme Triggered
Dynamic Self-Assembly. Polymers.

[62] Ahmadi M., Seiffert S. (2021). Direct Evidence of
Heteroleptic Complexation in the
Macroscopic Dynamics of Metallo-supramolecular Polymer Networks. Macromolecules.

[63] Frisch H., Nie Y., Raunser S., Besenius P. (2015). pH-Regulated selectivity in supramolecular
polymerizations: switching between co-and homopolymers. Chem. —Eur. J..

[64] Cohen S. I., Linse S., Luheshi L. M., Hellstrand E., White D. A., Rajah L., Otzen D. E., Vendruscolo M., Dobson C. M., Knowles T. P. (2013). Proliferation of amyloid-β42
aggregates occurs through a secondary nucleation mechanism. Proc. Natl. Acad. Sci. U.S.A..

[65] Miller Y., Ma B., Nussinov R. (2010). Zinc ions
promote Alzheimer Aβ aggregation via
population shift of polymorphic states. Proc.
Natl. Acad. Sci. U.S.A..

[66] Ahmadi M., Seiffert S. (2020). Thermodynamic control
over energy dissipation modes
in dual-network hydrogels based on metal–ligand coordination. Soft Matter.

[67] Wegner S. V., Schenk F. C., Witzel S., Bialas F., Spatz J. P. (2016). Cobalt
cross-linked redox-responsive PEG hydrogels: From viscoelastic liquids
to elastic solids. Macromolecules.

[68] Harris R. D., Auletta J. T., Motlagh S. A. M., Lawless M. J., Perri N. M., Saxena S., Weiland L. M., Waldeck D. H., Clark W. W., Meyer T. Y. (2013). Chemical and electrochemical manipulation of mechanical
properties in stimuli-responsive copper-cross-linked hydrogels. ACS Macro Lett..

[70] Zandomeneghi G., Krebs M. R., McCammon M. G., Fändrich M. (2004). FTIR reveals
structural differences between native β-sheet proteins and amyloid
fibrils. Protein Sci..

[71] Saini A., Rai S., Maiti D., Dutta A. (2022). Exploring the Cobalt–Histidine
Complex for Wide-Ranging Colorimetric O2 Detection. ACS Omega.

