# Supplementary material for: Metal Ion-Specific Modulation of Network Connectivity and Defects in Poly(ethylene glycol)–Peptide Conjugate Assemblies and Hydrogels
Source: Chem Mater. 2026 Jan 15;38(3):1240–52. doi: 10.1021/acs.chemmater.5c02542 (PMC12895393; doi:10.1021/acs.chemmater.5c02542)
Supplement: Supplementary file 1 [file cm5c02542_si_001.pdf]

# - Supporting Information -

Metal Ion-Specific Modulation of Network Connectivity and Defects in Poly(ethylene glycol)–Peptide Conjugate Assemblies and Hydrogels

Mostafa Ahmadi, Kamila Wittek, Hanna Sophie Rieger, Marius Thomas, Lars Hartmann, Pol Besenius,*

Sebastian Seiffert*

Department of Chemistry, Johannes Gutenberg-Universität Mainz, Duesbergweg 10-14, D-55128 Mainz, Germany

S1. Synthesis and Characterization

General Considerations

All solvents and chemicals used for synthesis were purchased from commercial sources and used without further purification if not stated otherwise. Reagents used for peptide synthesis were used in peptide grade quality. Polyethylene glycole diamine was purchased from Rapp Polymere (Tübingen, Germany). All reactions were performed in inert atmosphere. Data was plotted using Origin 2022 by OriginLab if not statet otherwise.

Gel permeation Chromatography (GPC)

Gel permeation chromatography (GPC) was performed on an Agilent 1260 Infinity II Series from Agilent Technologies (Waldbronn, Germany) in HFIP containing 3 g/L KTFA at 40°C using a two PFG column system (100/1000 Å porosity) from PSS Polymer Standards Services (Mainz, Germany). An RI detector was employed for detection of the samples. Polyethylene glycole standards from PSS PSS Polymer Standards Services were used for calibration. The obtained elugramms were evaluated using PSS WinGPC Unichrom.

Preparative high performance liquid chromatography (prep. HPLC)

Preparative HPLC was performed using an 1260 Infinity II system from Agilent (Waldbronn, Germany) equipped with a UV detector. A VP250/31 phenyl-hexyl column with 5 µm particles fro Macherey Nagel was used as a stationary phase. The mobile phase consisted of ammonium acetate buffer (solvent A, 20 mM NH_4OAc with 0.1 v/v% HOAc) and ACN (solvent B). Flow rate was adjusted to 37.0 mL min^{-1} The UV detector monitored absorbance at 214 and 254 nm. The following gradient was used:

Time (min)	Solvent A (%)	Solvent B (%)
0	70	30
5	70	30
30	1	99

Nuclear magnetic resonance spectroscopy (NMR)

^{1}H-NMR spectra were measured using a Bruker Avance II 400 spectrometer (400 MHz) from Bruker (Rheinstätten, Germany). Deuterated DMSO (DMSO-d_6) was used for all spectra and purchased from Deutero (Kastellaun, Germany). The coupling constant J is given in Hertz (Hz) and the chemical shifts are given in parts per million (ppm) and are relative to the residual solvent peak. The multiplicity of the signals is given by the following abbreviations: s = singlet, d = doublet, t= triplet, q = quartet, p = pintet and m = multiplet. All NMR spectra were analyzed using MestreNova 14.3.2 by MestreLab Research S.L. (Santiago de Compostela, Spain).

Matrix-assisted laser desorption ionization spectrometry (MALDI)

Matrix-assisted laser desorption ionization mass spectrometry with time of flight (ToF) analysis was performed on an Autoflex maX instrument by Bruker (Rheinstätten, Germany). All samples were measured in linear mode. The samples were prepared at a concentration of 1 mg mL^{-1}. DCTB with addition of KTFA was used as a matrix. Data acquisition and data analysis was carried out with Bruker flexControl 3.4 and flexAnalysis 3.4 by Bruker.

Compound 1: Pentapeptide

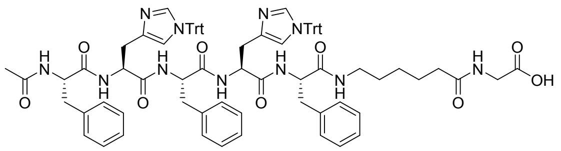

The synthesis was performed following literature known procedures using a solid phase peptide synthesizer. 2-Chlorotrityl chloride resin (loading capacity: 1.60 mmol/g, 1.0 eq.) was weighed into a peptide synthesis reactor and the first fmoc-protected amino acid (2.0 eq.) dissolved in DCM (10.0 mL / g resin) was added. After the addition of DIPEA (2.0 eq.), the reactor was shaken for 5 minutes at room temperature. Another portion of DIPEA (3.0 eq.) was added to the solution and the reactor shaken for another hour. MeOH (15.0 mL) were added afterwards and the reactor shaken for 15 more minutes. The solution was then drained and the resin washed consecutively with DCM, DMF, DCM and MeOH.

After swelling the resin in DCM for 10 minutes and draining the solvent, piperidine (20 v/v% in DMF) was added to the resin and mixed with the resin for 20 minutes. The solution was drained and the resin washed thoroughly with DMF. This process was repeated twice.

Solutions of the respective fmoc-protected amino acid (4.0 eq.), HOBt (4.0 eq.), HBTU (4.0 eq.) and DIPEA (4.0 eq.) in DMF were premixed before being transferred into the reactor. The reactor was then mixed for 1 hour. The solutions were then drained and washed consecutively with small portions of DMF.

This cycle was repeated for the coupling of every amino acid. After the cleavage the fmoc-protecting group on the last amino acid, an *N*-acetylation was performed using a capping solution consisting of acetic anhydride (0.5 M), DIPEA (0.125 M) and HOBt (0.015 M). The resin was washed with DCM afterwards.

The cleavage of the peptide was performed by mixing the resin with a solution of TFE (20 v/v% in DCM) and shaken at room temperature for 1 hour. The solution was then drained and the resin washed with DCM. The so obtained solution was evaporated in vacuo and codistilled with toluene. The residual solid was then lyophilized to obtained the desired peptide as amorphous solid.

Yield: 1.83 g (1.28 mmol, 80%), colorless solid.

MF $C_{87}H_{87}N_{11}O_9$ **MW** 1400 g mol^{-1}

TOF-MS (ESI, pos.) m/z: 1430.6759 $[M+H]^+$ (calc. 1430.6761).

^{1}H-NMR (400 MHz, DMSO-d_6, 296 K, COSY) δ [ppm] 8.47 (d, *J* = 7.5 Hz, 1H), 8.19 (d, *J* = 7.5 Hz, 1H, N*H*), 8.11 – 7.99 (m, 3H, N*H*), 7.96 (t, *J* = 5.7 Hz, 1H, N*H*), 7.88 (d, *J* = 8.1 Hz, 1H, N*H*), 7.38 – 7.28 (m, 19H, $CH^{Phe,Trityl}$), 7.20 – 7.01 (m, 28H, ^{Phe,Trityl,His}), 6.68 (s, 1H, CH^{His}), 6.58 (s, 1H, CH^{His}), 4.46 – 4.32 (m, 5H, α-$CH^{Phe,His}$), 3.71 (d, *J* = 5.7 Hz, 2H, α-CH^{Gly}), 3.05 – 2.60 (m, 12H, β- $CH_2^{Phe,His,Ahx}$), 2.05 (t, *J* = 7.5 Hz, 2H, CH_2^{Ahx}), 1.67 (s, 3H, CH_3), 1.40 (p, *J* = 7.6 Hz, 2H, CH_2^{Ahx}), 1.24 (q, *J* = 6.9 Hz, 2H, CH_2^{Ahx}), 1.11 (td, *J* = 8.5, 4.0 Hz, 2H, CH_2^{Ahx}).

Compound 2: PEG–Peptide conjugate

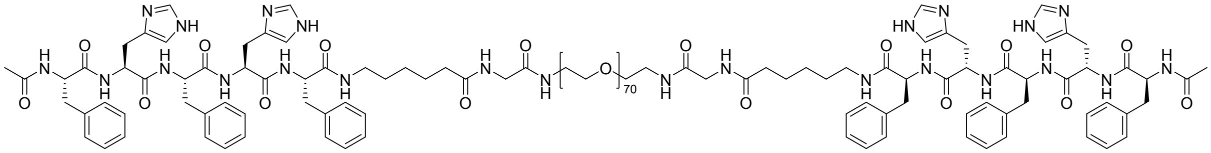

The peptide (97.0 mg, 0.068 mmol, 2.4 eq.), PyBOP (36.0 mg, 0.071 mmol, 2.5 eq.) and were dissolved in DMF (1.0 mL) and cooled to 0°C while stirring. DIPEA (14 µL, 0.079 mmol, 2.8 eq.) in DMF (0.5 mL) was added dropwise to the reaction mixture and the solution was stirred for 5 minutes. PEG diamine (Mn = 3000 g mol^{-1}, 85.0 mg, 0.028 mmol, 1.0 eq.) dissolved in DMF (0.5 mL) was added and the mixture was stirred for 2 hours at room temperature, before another portion of PyBOP (18.0 mg, 0.036 mmol, 1.3 eq.) and DIPEA (5.0 µL, 0.026 mmol, 0.9 eq.) was added. The reaction was then stirred for further 16 hours. All volatiles were removed in vacuo and size exclusion chromatography (BioBeads, DMF) was performed to remove residual peptide and coupling agents. The crude product was treated with a cleavage cocktail (2.0 mL, TFA:TIPS:H_2O 95 : 2.5 : 2.5) for 45 min. After codistillation with toluene (3 x 10 mL), the crude was dissolved in DCM (3.0 mL) and precipitated from ice cold diethyl ether. The solid obtained after centrifugation and drying was subjected to HPLC for final purification.

Yield: 53.0 mg (0.010 mmol, 35%), colorless solid.

MF for n = 70 $C_{240}H_{402}N_{24}O_{86}$ **MW** 5000 g mol^{-1}

TOF-MS (MALDI, LP) (DCTB+KTFA) m/z: 5037.8802 $[M+K]^+$ (calc. 5037.7520) for n=70.

^{1}H-NMR (400 MHz, DMSO-d_6, 296 K, COSY) δ [ppm] 11.85 (s, 4H, NH^{His}), 8.44 (s, 2H, NH^{amide}), 8.28 – 7.97 (m, 12H, NH^{amide}), 7.87 (t, *J* = 5.6 Hz, 2H, , NH^{Ahx}), 7.55 (s, 2H, CH^{His}), 7.51 (s, 2H, CH^{His}), 7.29 – 7.10 (m, 30H, CH^{Phe}), 6.78 (s, 4H, CH^{His}), 4.51 – 4.34 (m, 10H, α-C$H^{Phe,His}$), 3.66 (d, *J* = 5.8 Hz, 4H, α-CH^{Gly}), 3.51 (s, 280H, CH_2-CH_2^{PEG}), 3.21 (m, 4H, NH-CH_2^{Ahx}), 3.09 – 2.65 (m, 24H, β-C$H_2^{Phe,His,Ahx}$), 2.10 (t, *J* = 7.5 Hz, 4H, CO-CH_2^{Ahx}), 1.76 (s, 6H, CH_3^{Ac}), 1.47 (p, *J* = 7.6 Hz, 4H, CH_2^{Ahx}), 1.40 – 1.31 (m, 4H, CH_2^{Ahx}), 1.27 – 1.14 (m, 4H, CH_2^{Ahx}).

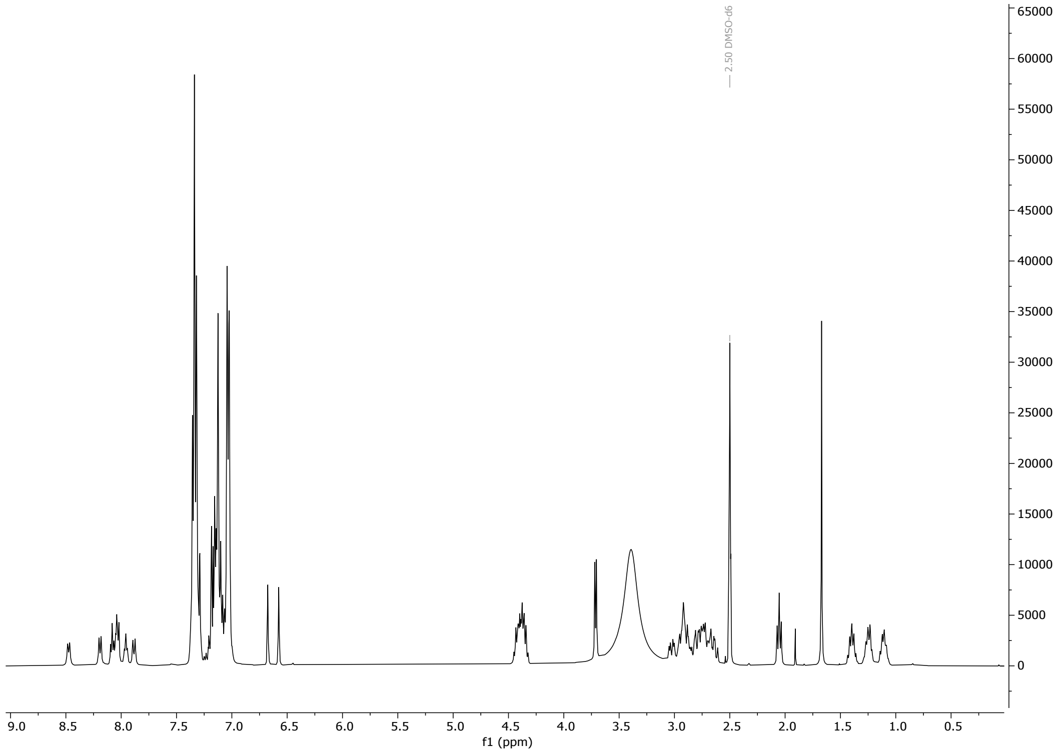

Figure S1. ^{1}H-NMR of Compound 1.

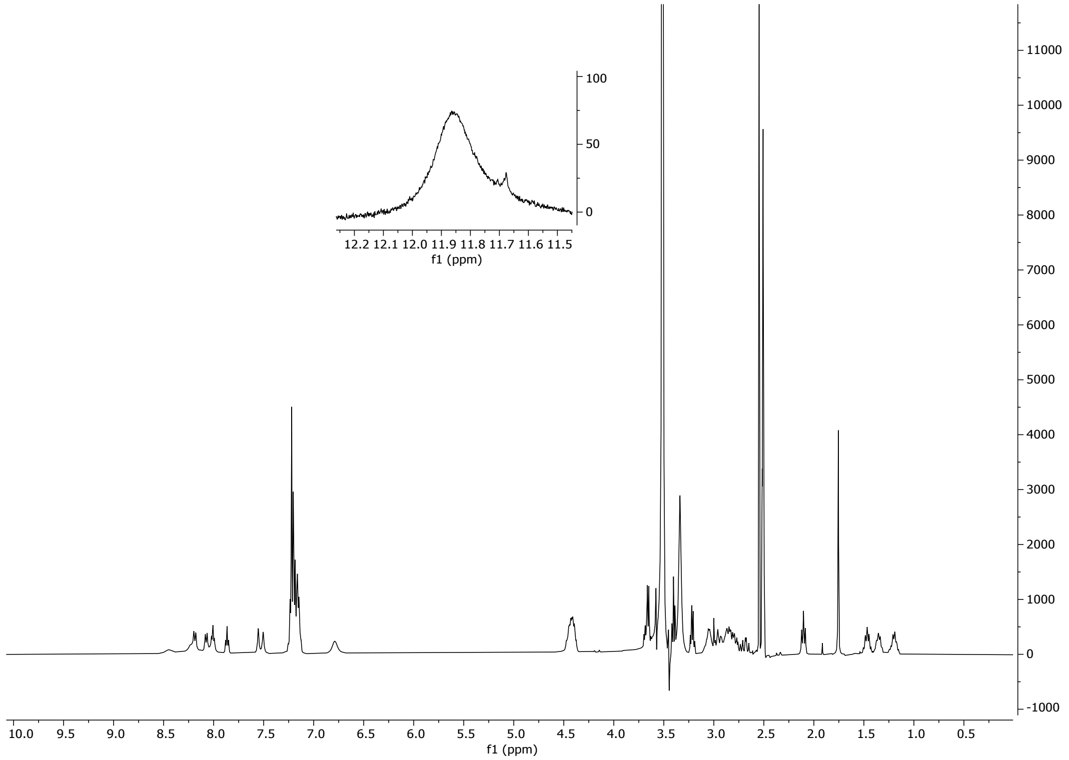

Figure S2. ^{1}H-NMR of Compound 2.

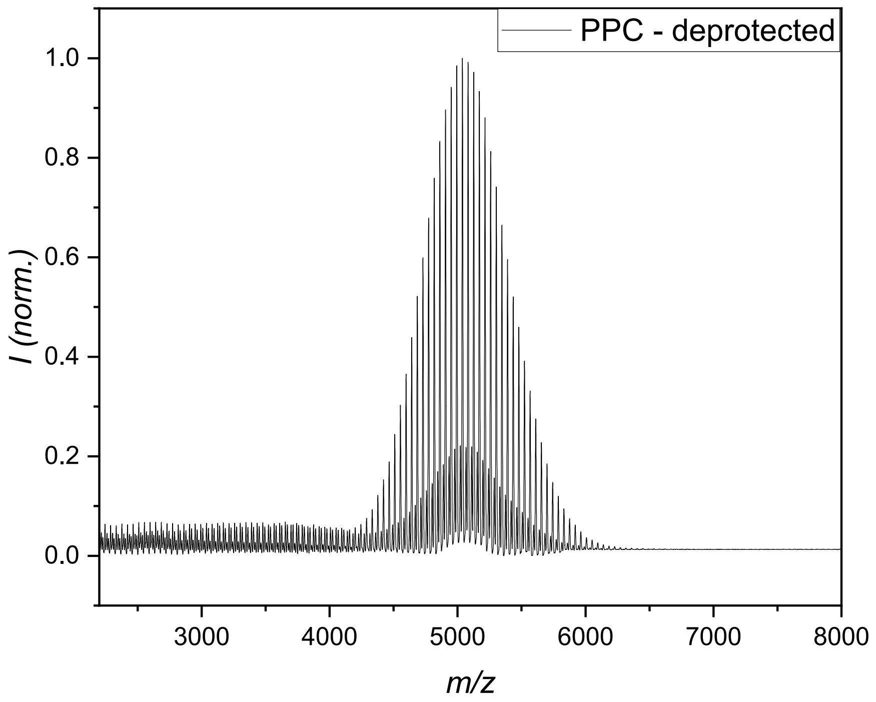

Figure S3. MALDI-ToF of Compound 2.

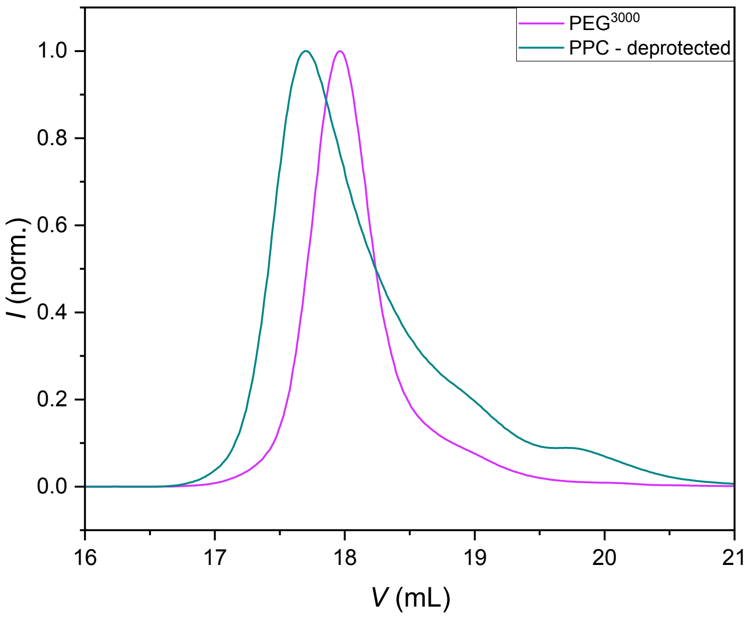

Figure S4. GPC elugramms of Compound 2 and PEG diamine in comparison.

S2. Analysis of the plain PEG-peptide

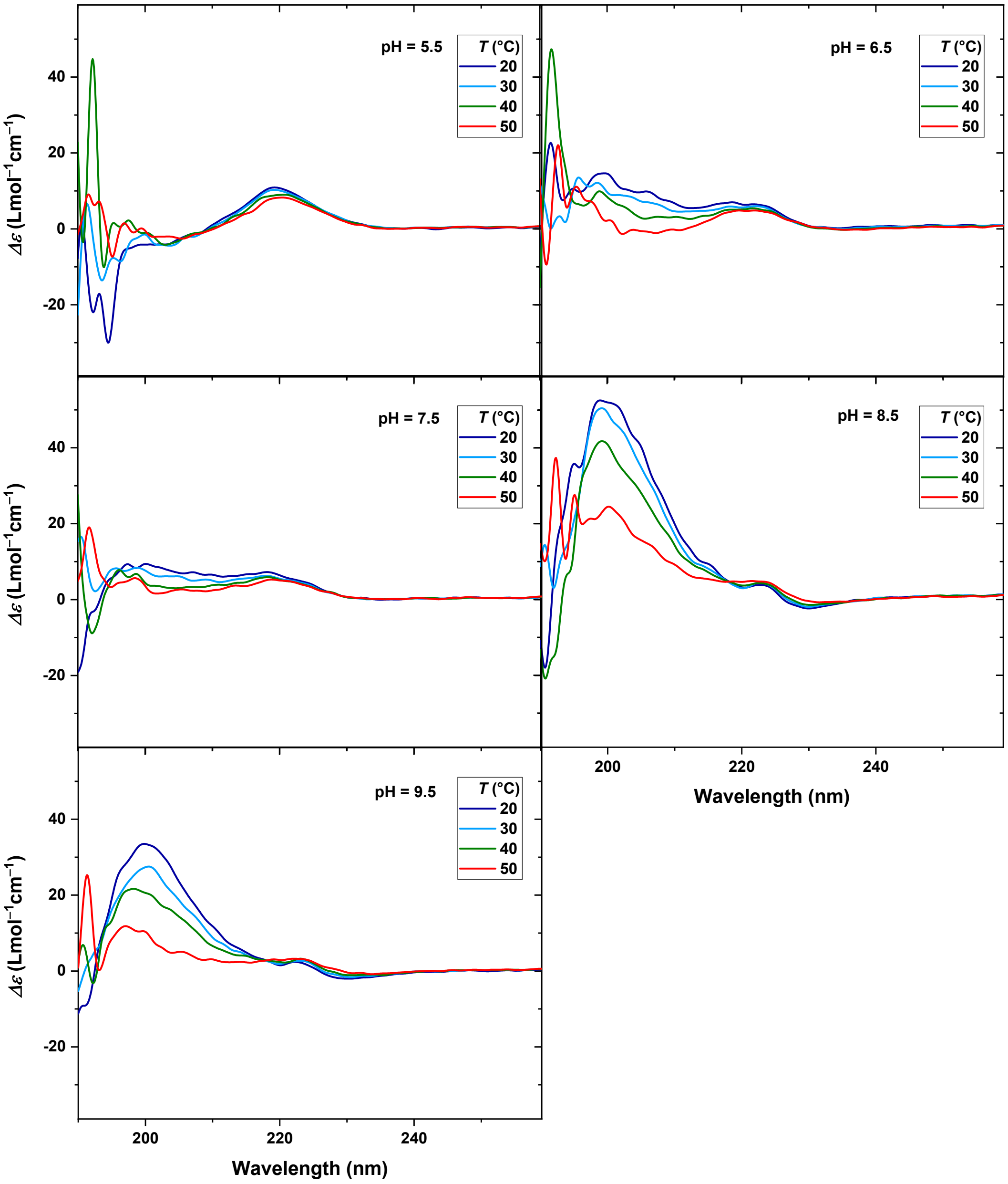

Figure S5. CD spectra of the plain PEG-peptide conjugate as a function of pH and temperature, as indicated in the legends.

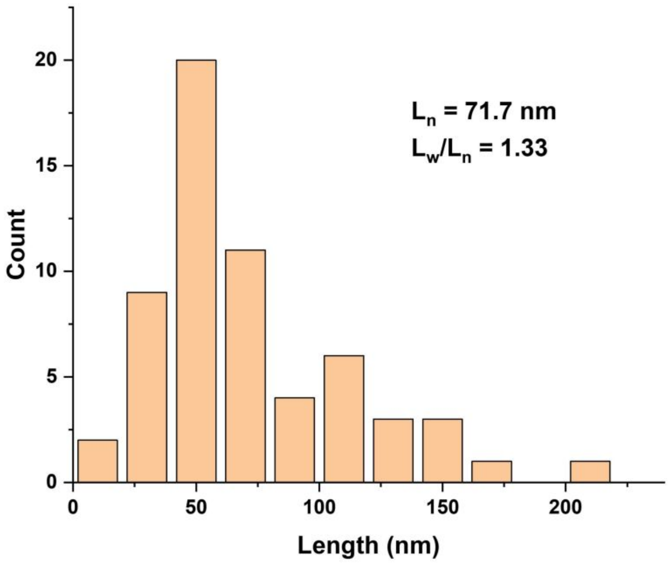

Figure S6. Histogram of nanofiber length of the plain PEG-peptide conjugate at φ = 2 wt% and pH = 7.5.

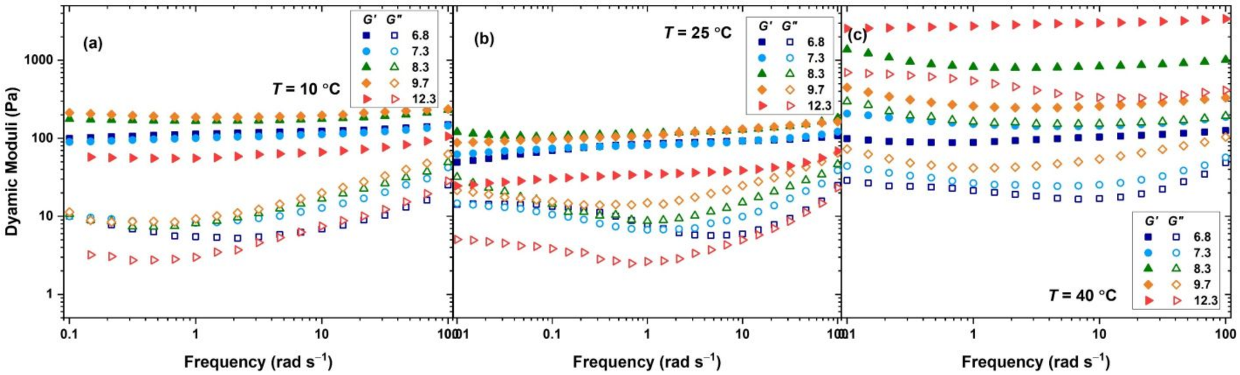

Figure S7. Dynamic storage (filled symbols) and loss (open symbols) moduli of hydrogels (φ = 2 wt%) at various pH (indicated in legends) and temperatures: (a) 10 °C, (b) 25 °C, (c) 40 °C (γ = 1%, *T* = 25 °C).

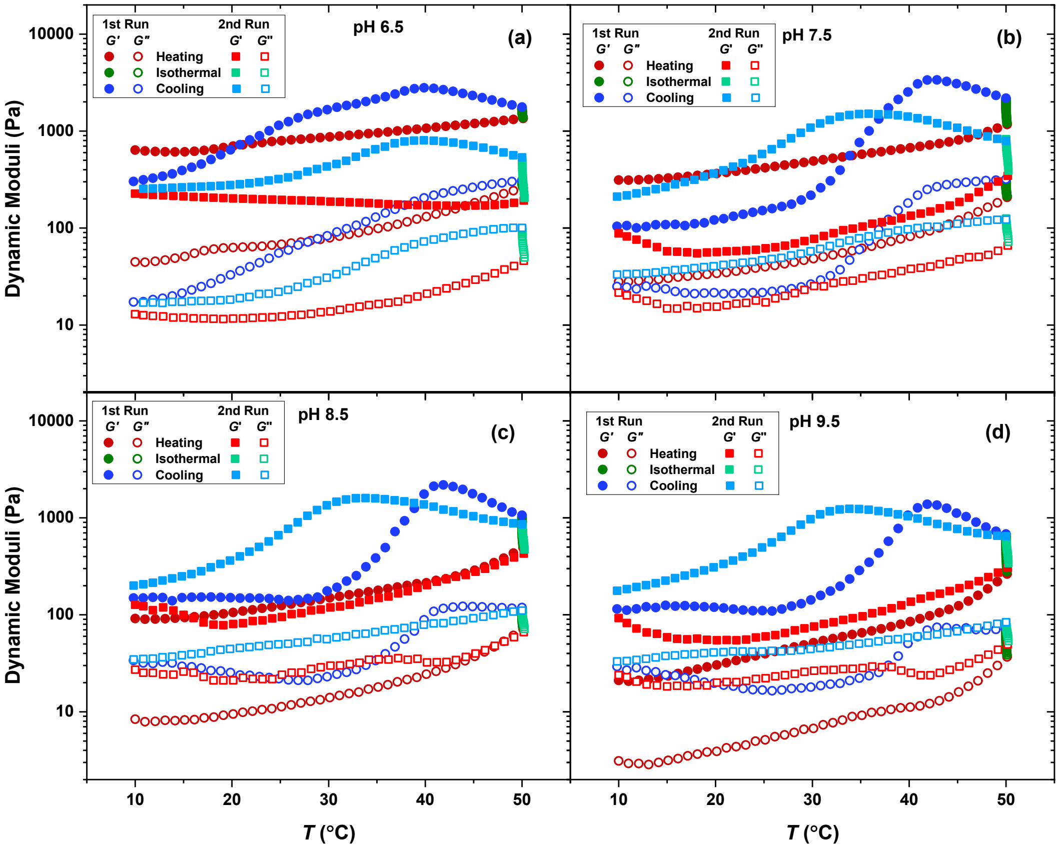

Figure S8. Oscillatory thermal treatment (γ = 1%, ω = 10 rad s^{-1}) including subsequent heating, isothermal, and cooling segments at rates of 0.02 (1st Run) and 0.04 C min^{-1} (2nd Run) for hydrogels at various pH values: (a) 6.5, (b) 7.5, (c) 8.5, (d) 9.5.

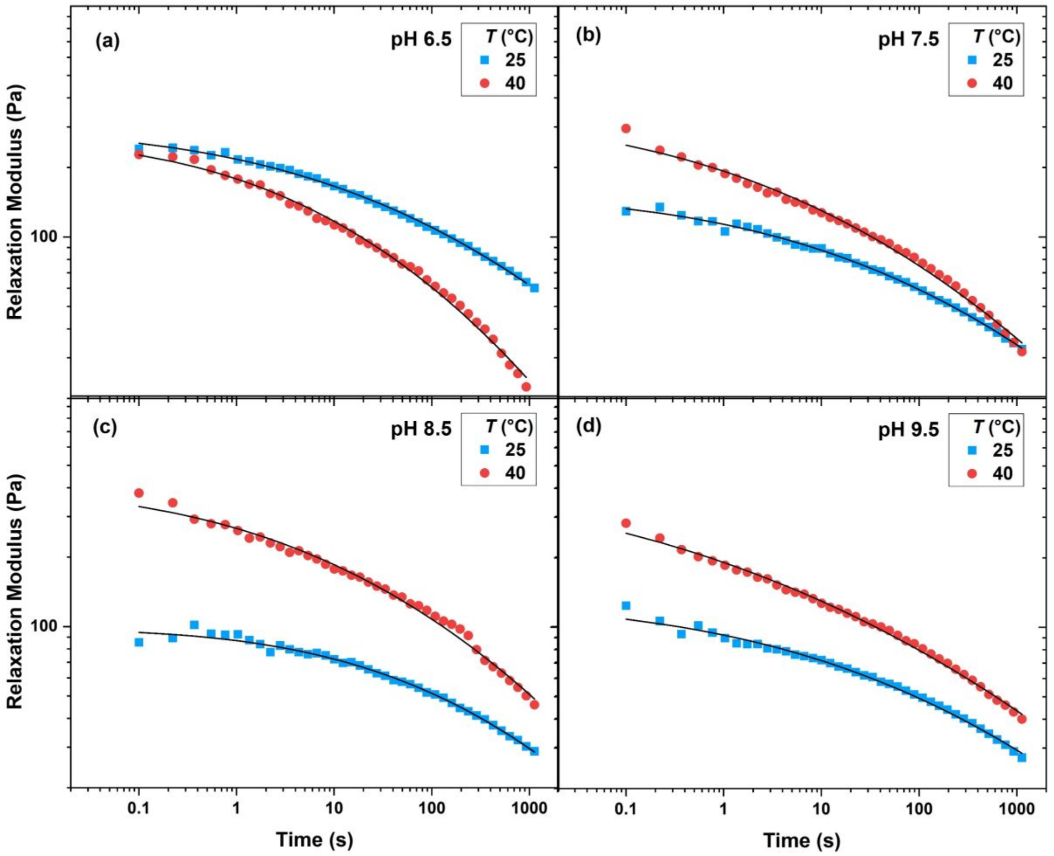

Figure S9. Relaxation modulus (symbols) and fit of the generalized Maxwell model (lines) for hydrogels at various pH values (φ = 2.0 g L^{-1}): (a) 6.5, (b) 7.5, (c) 8.5, (d) 9.5.

Table S1. Fit parameters of the generalized Maxwell model including the relaxation time, τ, standard deviation, σ, and plateau modulus, G_N^0 for hydrogels at φ = 2 wt% and listed pH and *T* values.

pH	*T* (°C)	τ (s)	σ	G_N^0 (Pa)
6.5	25	39.67	1.99	292.29
6.5	40	9.24	1.61	268.31
7.5	25	45.10	2.04	152.09
7.5	40	4.43	2.04	334.99
8.5	25	219.83	1.65	98.19
8.5	40	11.58	1.84	400.50
9.5	25	41.45	2.16	126.92
9.5	40	0.65	2.63	438.56

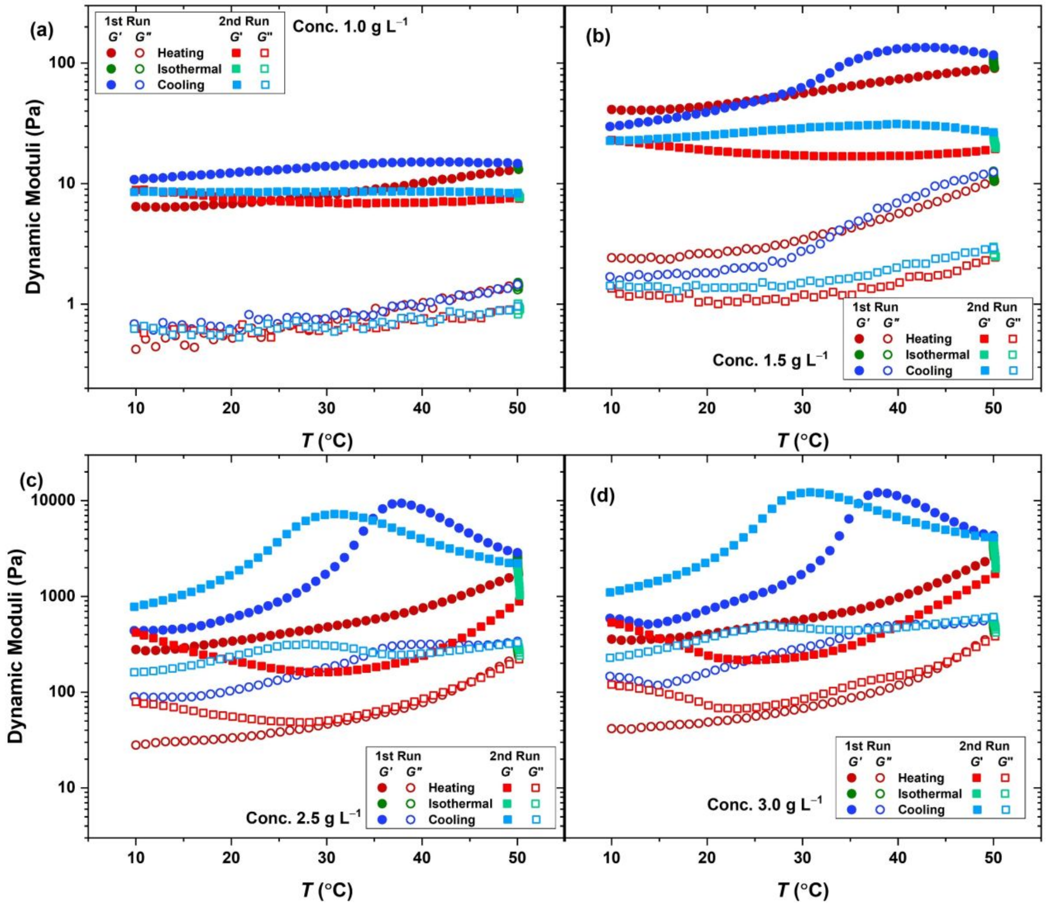

Figure S10. Oscillatory thermal treatment (γ = 1%, ω = 10 rad s^{-1}, pH = 7.5) including subsequent heating, isothermal, and cooling segments at rates of 0.02 (1st Run) and 0.04 C min^{-1} (2nd Run) for hydrogels at various concentrations: (a) 1.0 wt%, (b) 1.5 wt%, (c) 2.5 wt%, (d) 3.0 wt%.

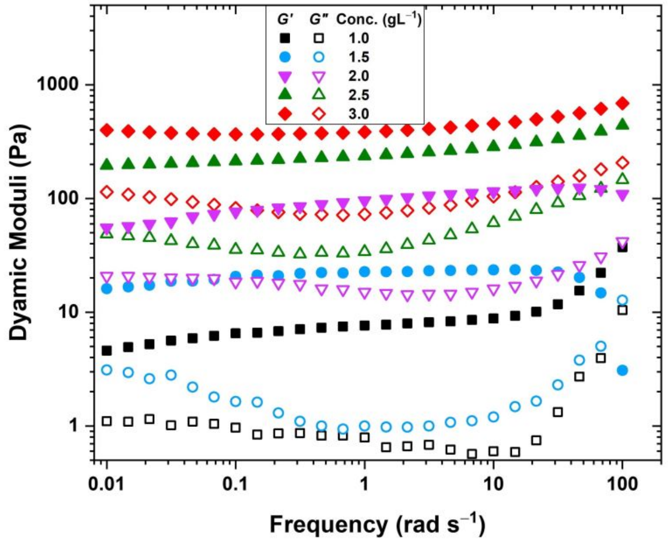

Figure S11. Dynamic storage (filled symbols) and loss (open symbols) moduli of hydrogels at various concentrations (γ = 1%, pH = 7.5, *T* = 25 °C).

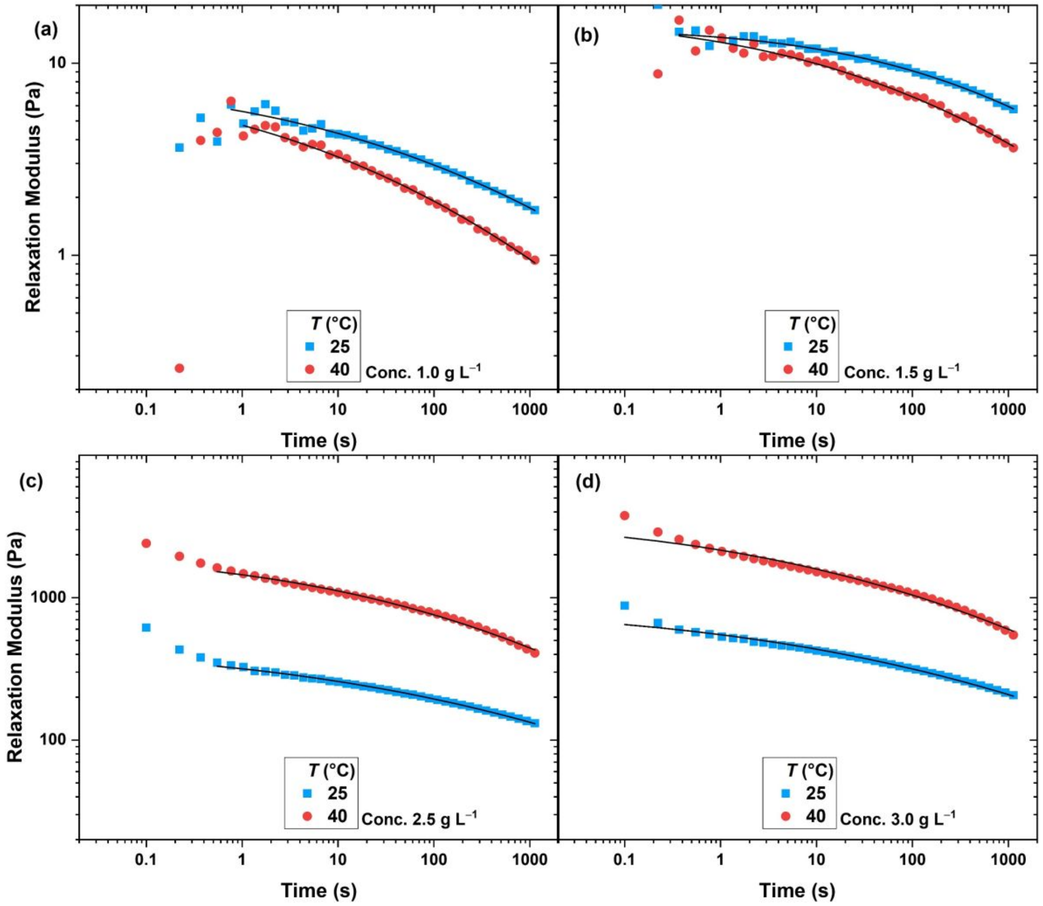

Figure S12. Relaxation modulus (symbols) and fit of the generalized Maxwell model (lines) of hydrogels at various concentrations (pH = 7.5): (a) 1.0, (b) 1.5, (c) 2.5, (d) 3.0 g L^{-1}.

Table S2. Fit parameters of the generalized Maxwell model including the relaxation time, τ, standard deviation, σ, and plateau modulus, G_N^0 for hydrogels at pH = 7.5 and listed concentration and *T* values.

Conc. (g L^{-1})	*T* (°C)	τ (s)	σ	G_N^0 (Pa)
1.0	25	31.37	2.24	7.95
1.0	40	4.67	2.09	8.22
1.5	25	577.98	1.78	14.98
1.5	40	48.97	1.99	16.97
2.0	25	45.10	2.04	152.09
2.0	40	4.43	2.04	334.99
2.5	25	78.32	2.70	443.99
2.5	40	39.18	2.12	1983.61
3.0	25	29.52	2.70	827.47
3.0	40	10.00	2.34	3466.72

S3. Analysis of PEG-peptide conjugate in the presence of Zn^{2+} ions

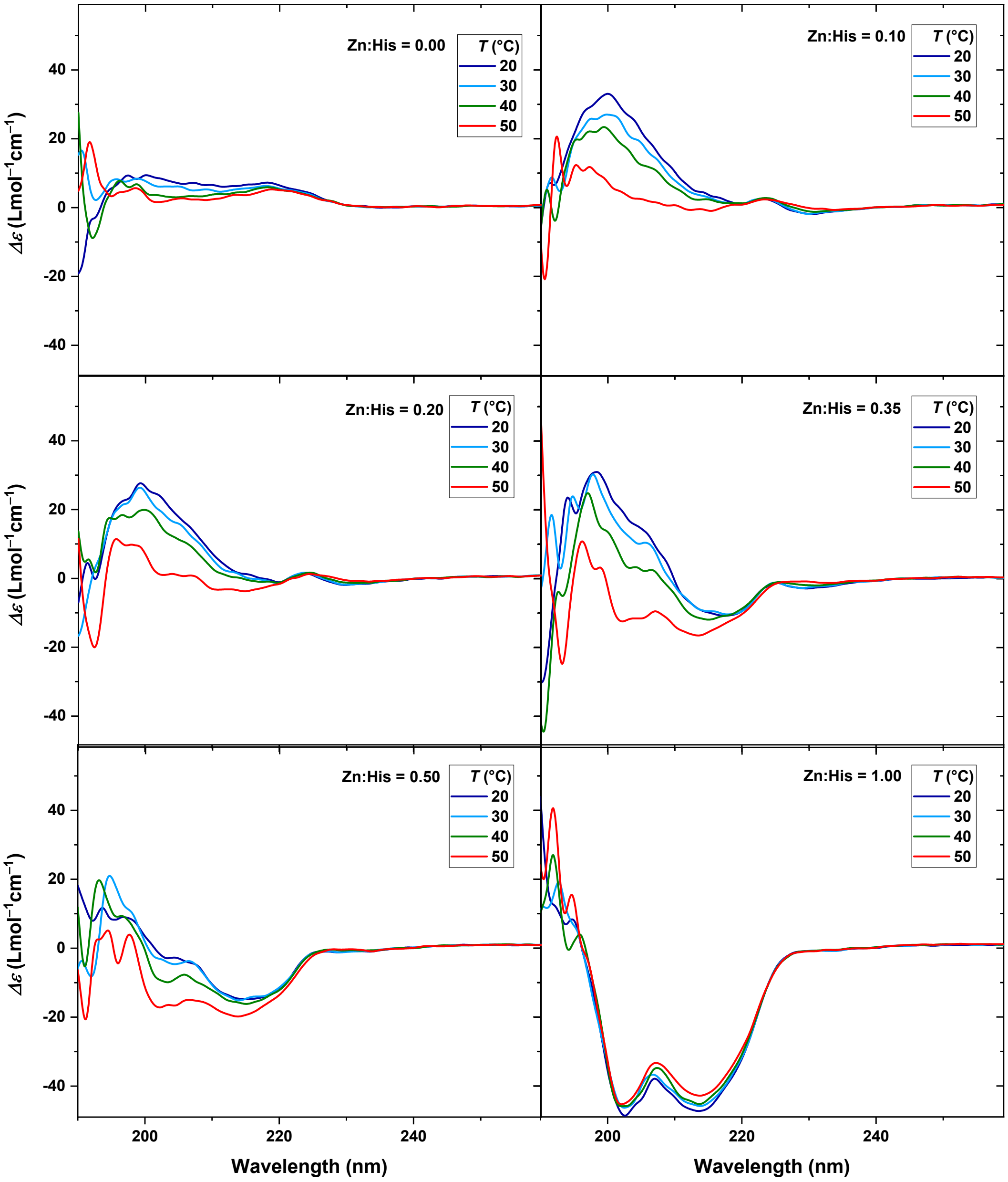

Figure S13. CD spectra of the PEG-peptide conjugate in the presence of Zn^{2+} at various Zn:His ratios, as indicated in the legends.

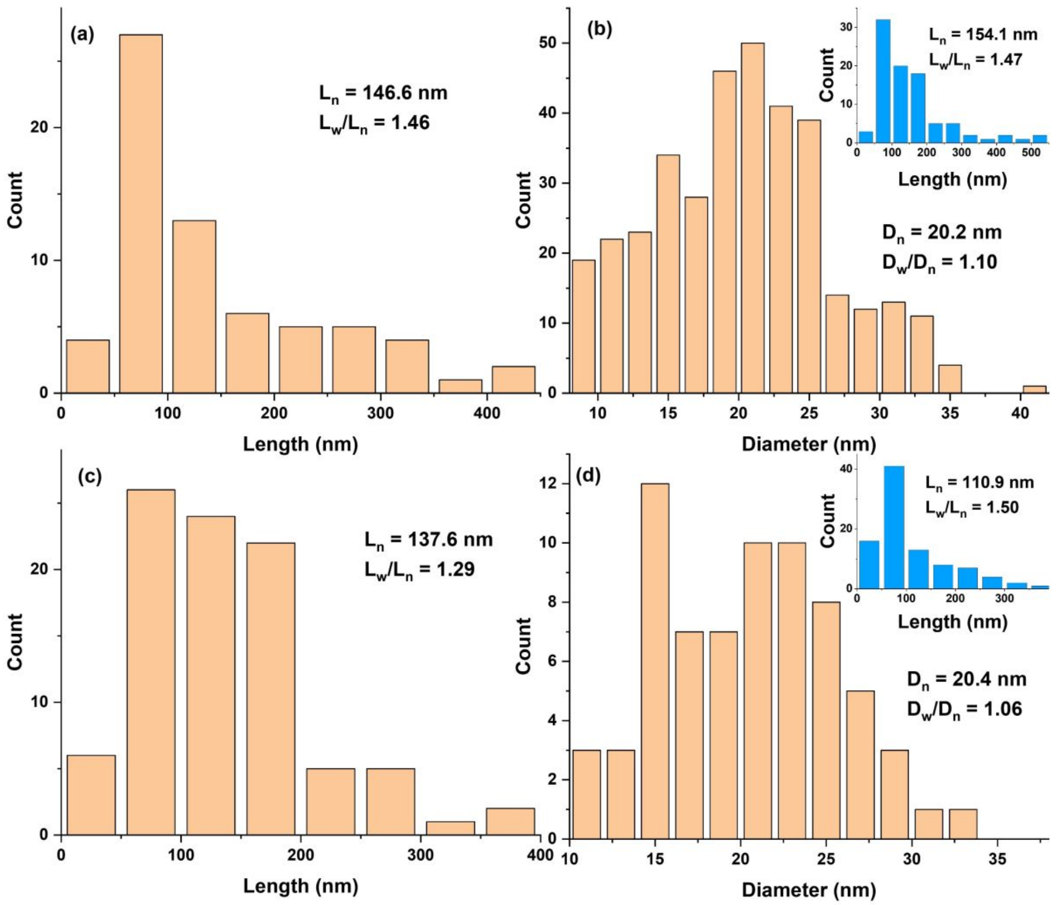

Figure S14. Histogram of nanofiber length and diameter of spherical particles at pH = 7.5 and various Zn:His ratios: (a) 0.1, (b) 0.2, (c) 0.5, (d) 1.0.

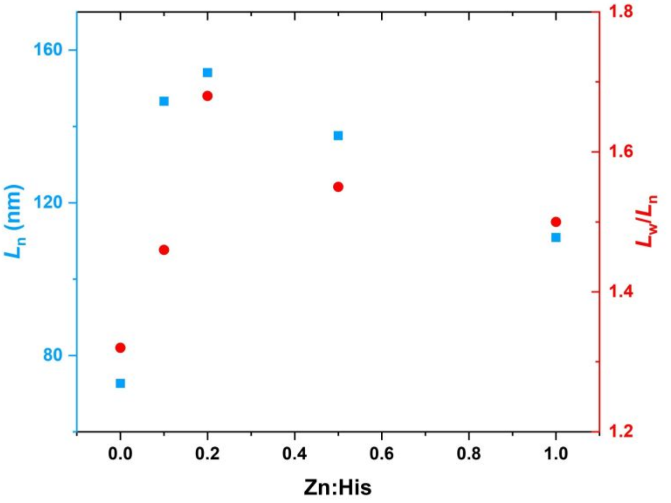

Figure S15. Number average length (left axis) and dispersity index (right axis) of nanofiber length as a function of Zn^{2+} content.

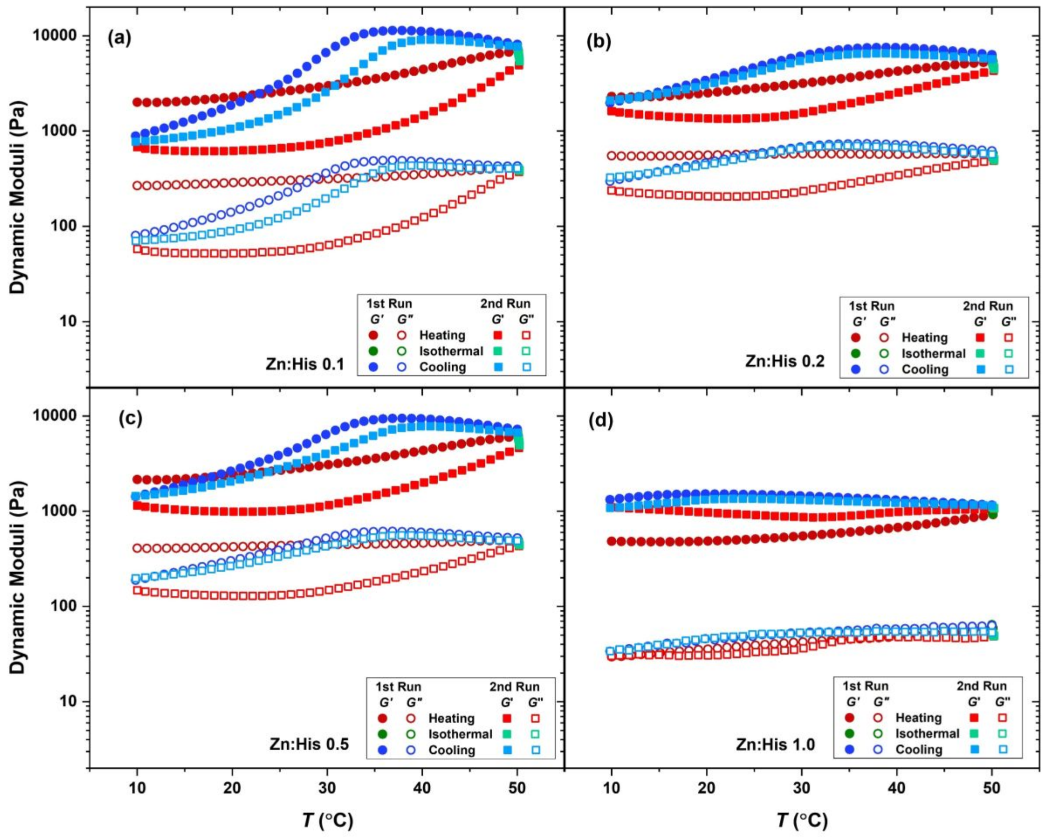

Figure S16. Oscillatory thermal treatment (γ = 1%, ω = 10 rad s^{-1}, pH = 7.5) including subsequent heating, isothermal, and cooling segments at rates of 0.02 (1st Run) and 0.04 C min^{-1} (2nd Run) for hydrogels at various Zn:His ratios: (a) 0.1, (b) 0.2, (c) 0.5, (d) 1.0.

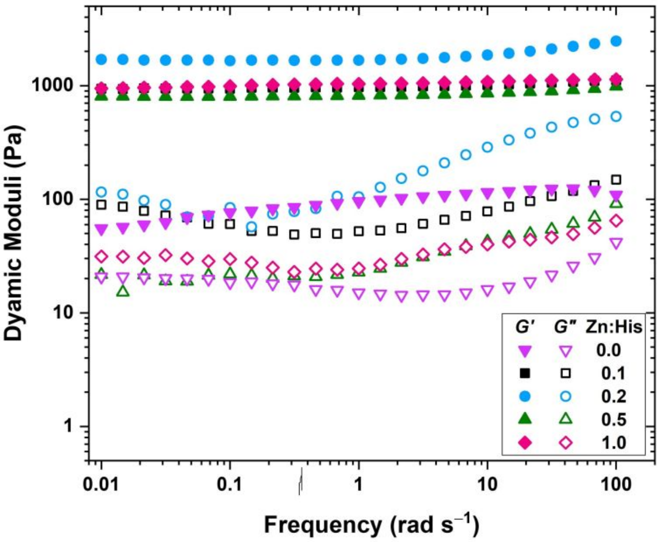

Figure S17. Dynamic storage (filled symbols) and loss (open symbols) moduli of hydrogels at various Zn:His ratios (γ = 1%, pH = 7.5, *T* = 25 °C).

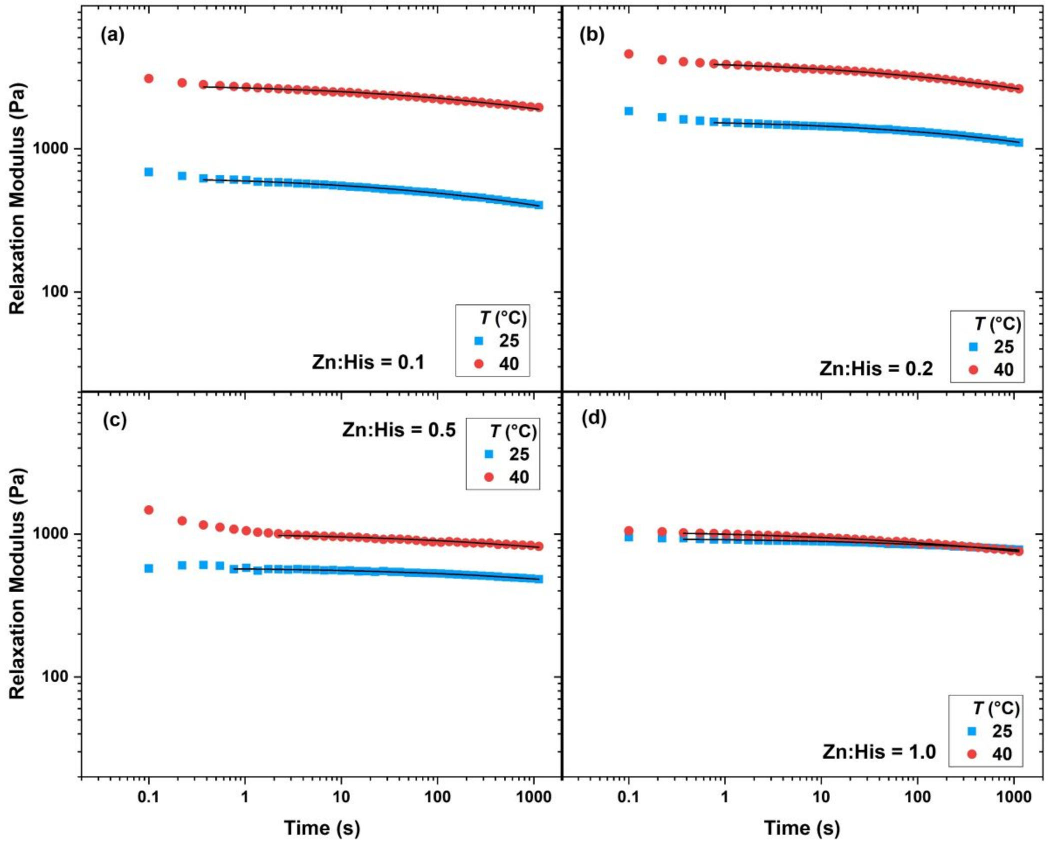

Figure S18. Relaxation modulus (symbols) and fit of the generalized Maxwell model (lines) of hydrogels at various Zn:His ratios: (a) 0.1, (b) 0.2, (c) 0.5, (d) 1.0.

Table S3. Fit parameters of the generalized Maxwell model including the relaxation time, τ, standard deviation, σ, and plateau modulus, G_N^0 for hydrogels at pH = 7.5, φ = 2 wt% and listed Zn:His ratios and *T* values.

Zn:His	*T* (°C)	τ (s)	σ	G_N^0 (Pa)
0	25	45.1	2.0	152.1
0	40	4.4	2.0	335.0
0.1	25	13677.1	2.5	639.6
0.1	40	28908.0	2.5	2817.1
0.2	25	41611.6	2.4	1582.6
0.2	40	15998.5	2.6	4155.8
0.5	25	813966.0	2.7	601.4
0.5	40	385250.7	2.7	1016.9
1	25	1071473.0	2.8	938.2
1	40	87995.8	2.8	1051.7

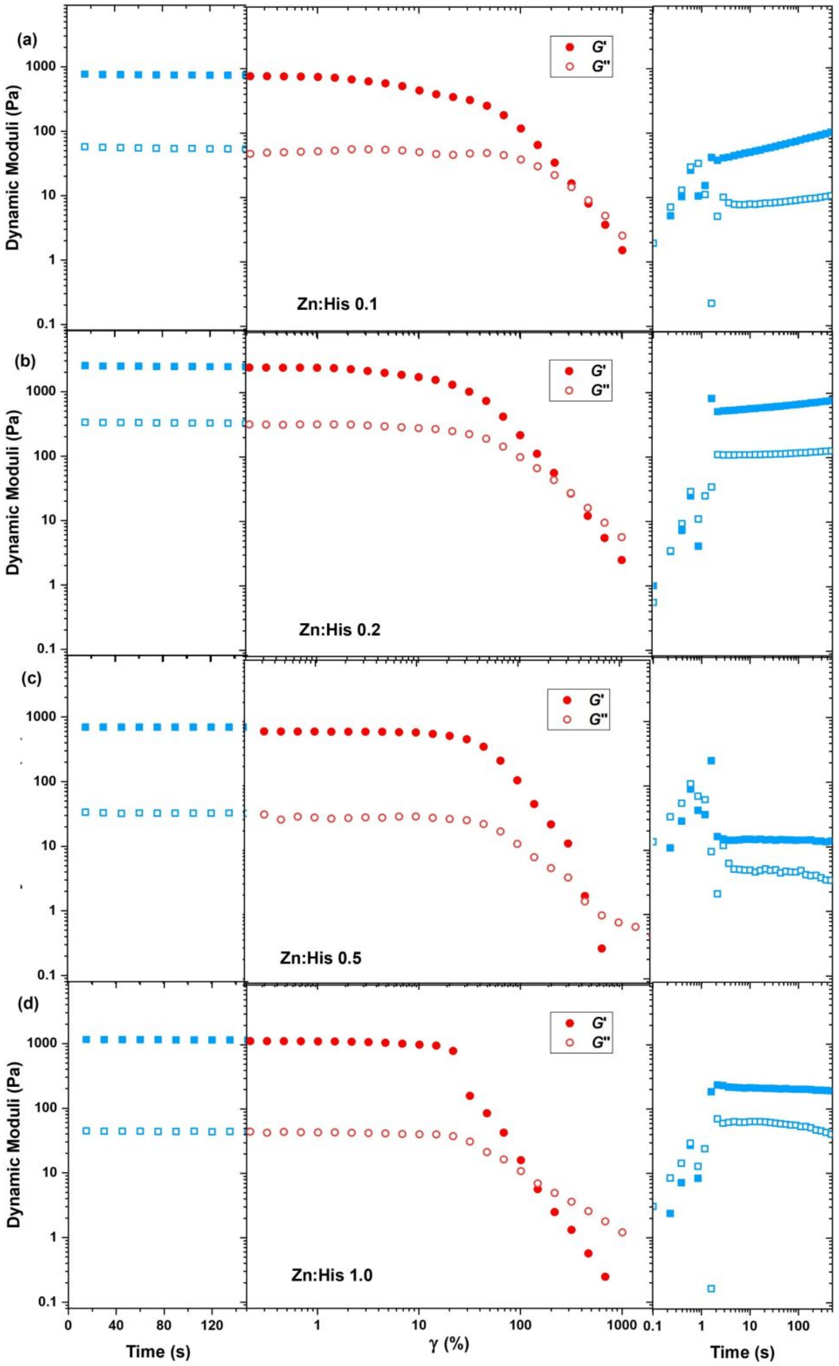

Figure S19. Dynamic storage (filled symbols) and loss (open symbols) moduli in network destruction and recovery, including time sweeps (γ = 1%, ω = 10 rad s^{-1}) before and after an amplitude sweep (ω = 10 rad s^{-1}) for hydrogels at various Zn:His ratios: (a) 0.1, (b) 0.2, (c) 0.5, (d) 1.0. (pH = 7.5, φ = 2.0 wt%, T = 25 °C).

S4. Analysis of PEG-peptide conjugate in the presence of other metal ions

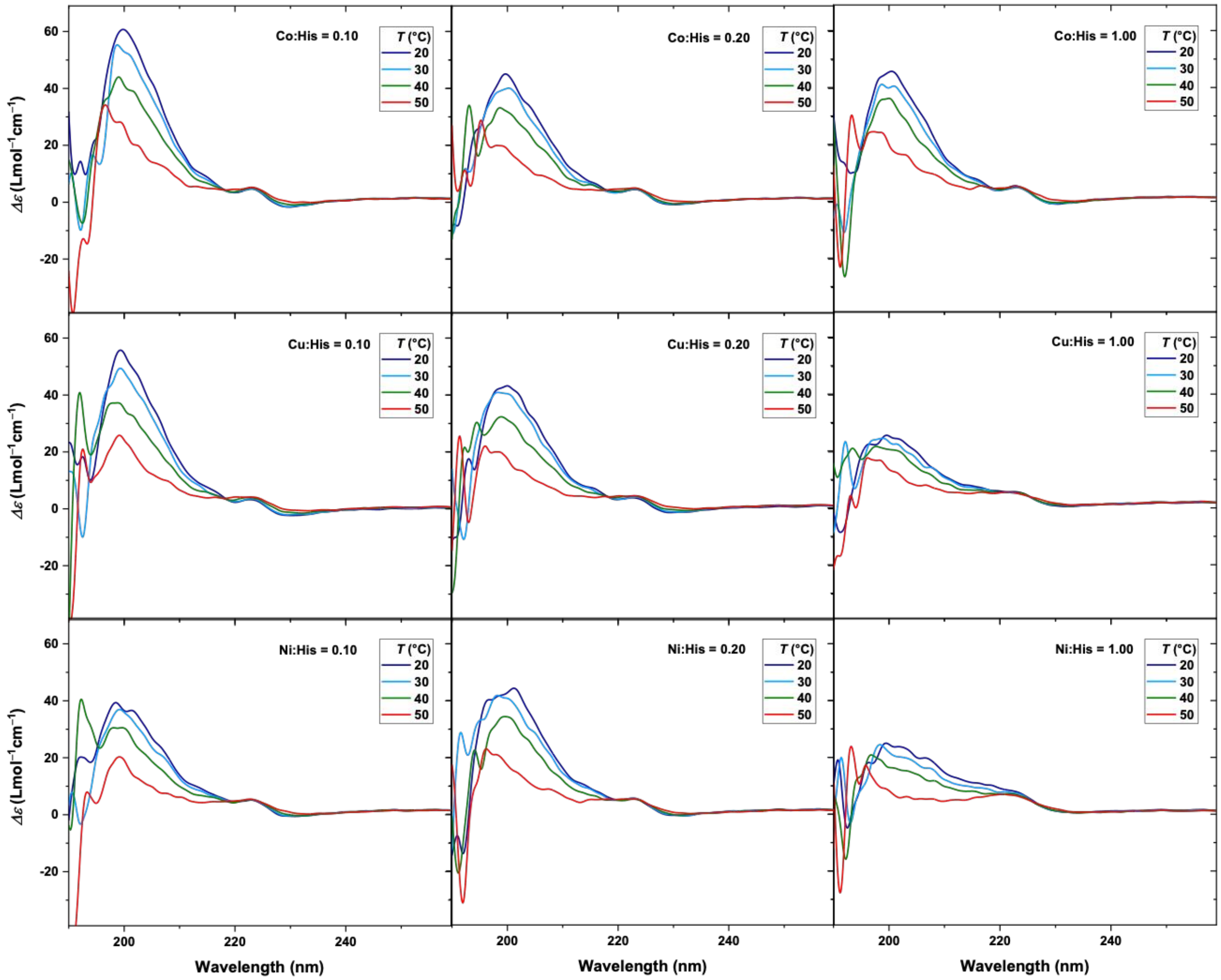

Figure S20. CD spectra of the PEG-peptide conjugate in the presence of various metal ions at different M:His ratios, as indicated in the legends.

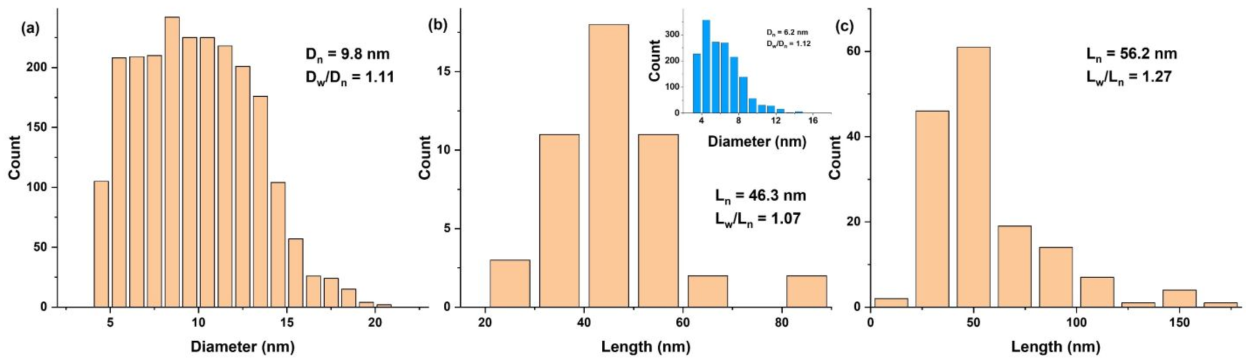

Figure S21. Histogram of nanofiber length and diameter of spherical particles at pH = 7.5 with various metal ions: (a) Co^{2+}, (b) Cu^{2+}, and (c) Ni^{2+} at M:His = 0.2.

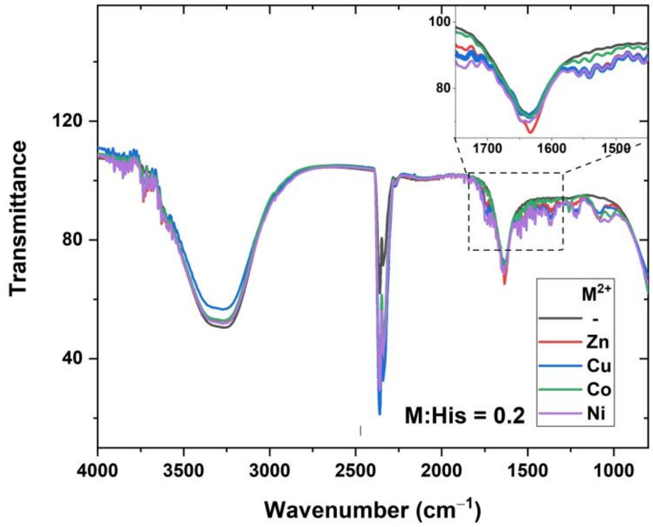

Figure S22. FTIR spectra of the PEG-peptide hydrogels obtained at pH = 7.5 in the presence of various metal ions.

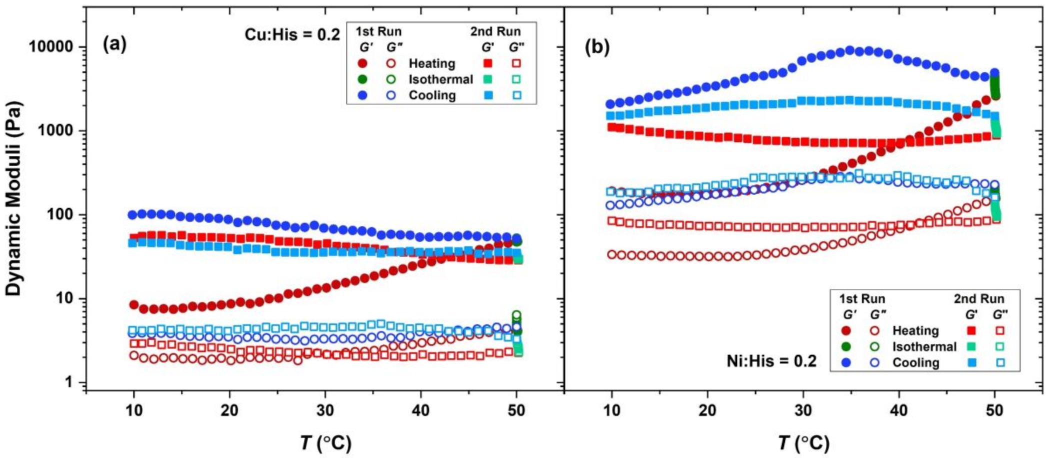

Figure S23. Oscillatory thermal treatment (γ = 1%, ω = 10 rad s^{-1}, pH = 7.5) including subsequent heating, isothermal, and cooling segments at rates of 0.02 (1st Run) and 0.04 C min^{-1} (2nd Run) for hydrogels obtained in the presence of (a) Cu^{2+} and (b) Ni^{2+} at M:His = 0.2.

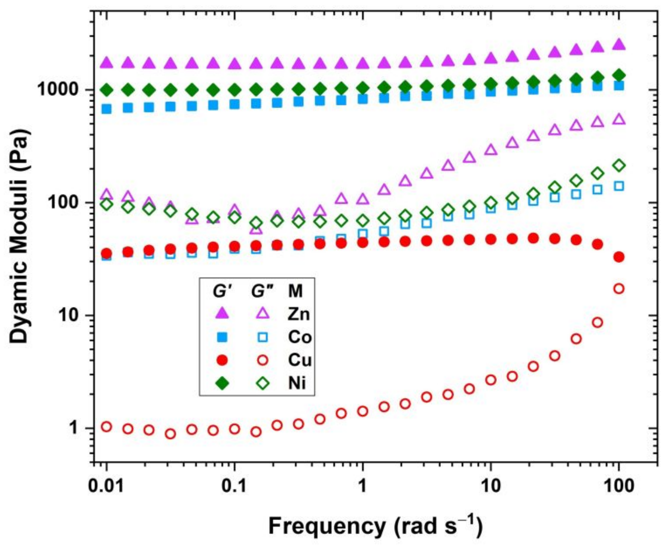

Figure S24. Dynamic storage (filled symbols) and loss (open symbols) moduli of hydrogels obtained in the presence of various metal ions (γ = 1%, pH = 7.5, M:His = 0.2, *T* = 25 °C).

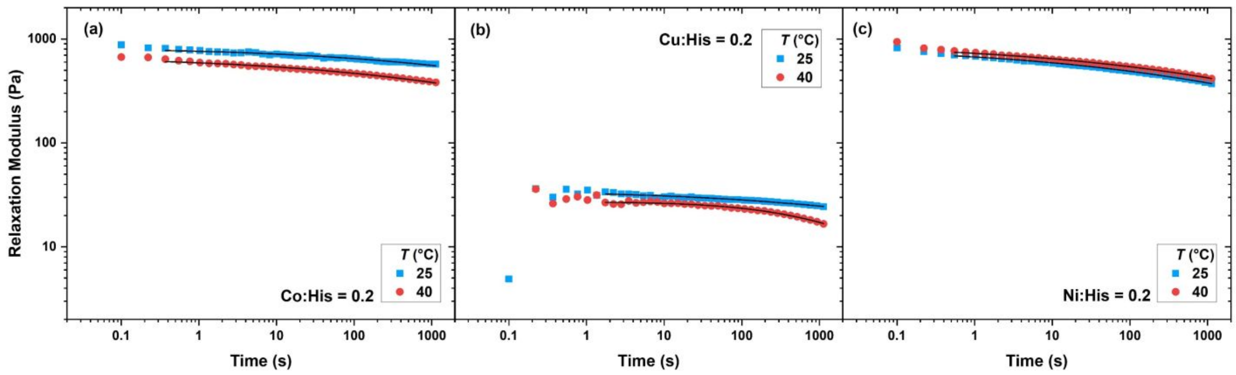

Figure S25. Relaxation modulus (symbols) and fit of the generalized Maxwell model (lines) of hydrogels obtained in the presence of various metal ions: (a) Co^{2+}, (b) Cu^{2+}, and (c) Ni^{2+} at M:His =0.2.

Table S4. Fit parameters of the generalized Maxwell model including the relaxation time, τ, standard deviation, σ, and plateau modulus, G_N^0 for hydrogels at pH = 7.5, φ = 2 wt%, at M:His = 0.2, and listed metal ions and *T* values.

M^{2+}	*T* (°C)	τ (s)	σ	G_N^0 (Pa)
-	25	45.10	2.04	152.09
-	40	4.43	2.04	334.99
Zn2+	25	41611.61	2.38	1582.55
Zn2+	40	15998.48	2.56	4155.84
Co^{2+}	25	42842.91	2.80	814.26
Co^{2+}	40	6604.35	2.80	660.33
Ni^{2+}	25	1074.36	2.80	805.08
Ni^{2+}	40	1613.70	2.80	856.67
Cu^{2+}	25	77487.45	2.80	34.48
Cu^{2+}	40	4707.94	1.15	27.07

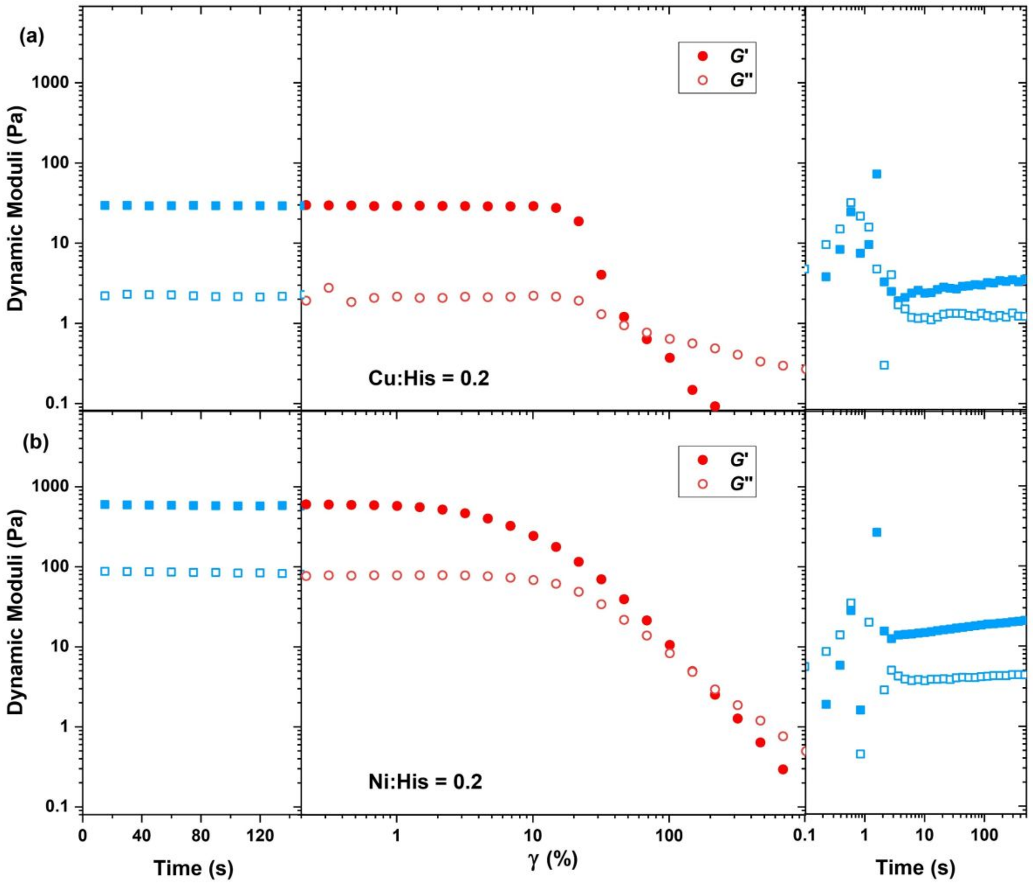

Figure S26. Dynamic storage (filled symbols) and loss (open symbols) moduli in network destruction and recovery, including time sweeps (γ = 1%, ω = 10 rad s^{-1}) before and after an amplitude sweep (ω = 10 rad s^{-1}) for hydrogels obtained in the presence of various metal ions: (a) Co^{2+}, (b) Cu^{2+}, and (c) Ni^{2+} (pH = 7.5, *φ* = 2.0 wt%, M:His =0.2, *T* = 25 °C).
